# A Temporal Gate for Viral Enhancers to Co-opt Toll-Like-Receptor Transcriptional Activation Pathways upon Acute Infection

**DOI:** 10.1371/journal.ppat.1004737

**Published:** 2015-04-09

**Authors:** Kai A. Kropp, Wei Yuan Hsieh, Elena Isern, Thorsten Forster, Eva Krause, Wolfram Brune, Ana Angulo, Peter Ghazal

**Affiliations:** 1 Division of Pathway Medicine, Edinburgh Infectious Diseases, University of Edinburgh, Edinburgh, United Kingdom; 2 Institut d’Investigacions Biomèdiques August Pi i Sunyer, University of Barcelona, Barcelona, Spain; 3 Heinrich Pette Institute, Leibniz Institute for Experimental Virology, Hamburg, Germany; 4 SynthSys, University of Edinburgh, The King’s Buildings, Edinburgh, United Kingdom; University of California, Los Angeles, UNITED STATES

## Abstract

Viral engagement with macrophages activates Toll-Like-Receptors (TLRs) and viruses must contend with the ensuing inflammatory responses to successfully complete their replication cycle. To date, known counter-strategies involve the use of viral-encoded proteins that often employ mimicry mechanisms to block or redirect the host response to benefit the virus. Whether viral regulatory DNA sequences provide an opportunistic strategy by which viral enhancer elements functionally mimic innate immune enhancers is unknown. Here we find that host innate immune genes and the prototypical viral enhancer of cytomegalovirus (CMV) have comparable expression kinetics, and positively respond to common TLR agonists. In macrophages but not fibroblasts we show that activation of NFκB at immediate-early times of infection is independent of virion-associated protein, M45. We find upon virus infection or transfection of viral genomic DNA the TLR-agonist treatment results in significant enhancement of the virus transcription-replication cycle. In macrophage time-course infection experiments we demonstrate that TLR-agonist stimulation of the viral enhancer and replication cycle is strictly delimited by a temporal gate with a determined half-maximal time for enhancer-activation of 6 h; after which TLR-activation blocks the viral transcription-replication cycle. By performing a systematic siRNA screen of 149 innate immune regulatory factors we identify not only anticipated anti-viral and pro-viral contributions but also new factors involved in the CMV transcription-replication cycle. We identify a central convergent NFκB-SP1-RXR-IRF axis downstream of TLR-signalling. Activation of the RXR component potentiated direct and indirect TLR-induced activation of CMV transcription-replication cycle; whereas chromatin binding experiments using wild-type and enhancer-deletion virus revealed IRF3 and 5 as new pro-viral host transcription factor interactions with the CMV enhancer in macrophages. In a series of pharmacologic, siRNA and genetic loss-of-function experiments we determined that signalling mediated by the TLR-adaptor protein MyD88 plays a vital role for governing the inflammatory activation of the CMV enhancer in macrophages. Downstream TLR-regulated transcription factor binding motif disruption for NFκB, AP1 and CREB/ATF in the CMV enhancer demonstrated the requirement of these inflammatory signal-regulated elements in driving viral gene expression and growth in cells as well as in primary infection of neonatal mice. Thus, this study shows that the prototypical CMV enhancer, in a restricted time-gated manner, co-opts through DNA regulatory mimicry elements, innate-immune transcription factors to drive viral expression and replication in the face of on-going pro-inflammatory antiviral responses in vitro and in vivo and; suggests an unexpected role for inflammation in promoting acute infection and has important future implications for regulating latency.

## Author Summary

Here we discover how inflammatory signalling may unintentionally promote infection, as a result of viruses evolving DNA sequences, known as enhancers, which act as a bait to prey on the infected cell transcription factors induced by inflammation. The major inflammatory transcription factors activated are part of the TLR-signalling pathway. We find the prototypical viral enhancer of cytomegalovirus can be paradoxically boosted by activation of inflammatory “anti-viral” TLR-signalling independent of viral structural proteins. This leads to an increase in viral gene expression and replication in cell-culture and upon infection of mice. We identify an axis of inflammatory transcription factors, acting downstream of TLR-signalling but upstream of interferon inhibition. Mechanistically, the central TLR-adapter protein MyD88 is shown to play a critical role in promoting viral enhancer activity in the first 6h of infection. The co-option of TLR-signalling exceeds the usage of NFκB, and we identify IRF3 and 5 as newly found viral-enhancer interacting inflammatory transcription factors. Taken together this study reveals how virus enhancers, employ a path of least resistance by directly harnessing within a short temporal window, the activation of anti-viral signalling in macrophages to drive viral gene expression and replication to an extent that has not been recognised before.

## Introduction

Infection by pathogens is detected by the host innate immune system through interaction of Pathogen-Associated Molecular Patterns (PAMPs) using a range of extra and intra-cellular host Pathogen-Recognitions-Receptors (PRRs) [1–3]. The major group of PRRs is represented by the family of Toll-Like-Receptors (TLRs) that detect a range of PAMPs and are located either at the cell surface, e.g. TLR2 and TLR4, or in endosomes, e.g. TLR3, 7 and 9 [3–5]. Binding of the corresponding ligands to these receptors leads to the activation of downstream signalling factors and TLR-receptors are dependent on the adaptor molecule MyD88, with exception of TLR3 and 4. TLR3 signals exclusively through the adaptor TRIF and TLR4 is the only TLR that can utilise both signalling pathways [3,6]. The activity of the TLR-signalling pathway triggers the expression of type I interferons and other antiviral factors that aid to control infections [7–9].

Cytomegalovirus (CMV) is recognised by the innate immune system using a diverse set of PRRs [1,10,11]. At the cell surface a direct interaction between the viral glycoproteins and TLR2 has been reported for human CMV (HCMV) [12,13] and also for the related human Herpesvirus 1 (HSV1) [14]. Other TLRs that play a role in resistance to CMV infection are TLR3 and TLR9. Homozygotic knockout animals for *Tlr2*, *Tlr3* or *Tlr9* are highly susceptible for CMV infection and show increased mortality rates [15,16]. Other types of PRRs have also been implicated to contribute to the detection of CMV infection. The cytoplasmic DNA sensors DAI (ZBP-1) [17] and AIM2 [18] have been shown to detect CMV. The interaction of viruses at the cell surface (TLR2), or intracellular recognition of viral genomes (by DAI, AIM2, TLR9) and virion packaged RNA (through RIG-I, TLR3, TLR7) [19] results in triggering anti-viral responses through the signal activation of downstream inflammatory transcription factors (TFs). Depending on the infected cell type, these signal regulated TFs include NFκB, AP1, CREB/ATF, IRF3 or IRF7 which govern the expression of pro-inflammatory and anti-viral host factors and effector molecules.

Virus genomes encode a number of proteins, termed evasins that help to evade and subvert the host immune response to the infection [20–23], many of which target molecules of the adaptive immune response. Some evasins, however, inhibit the innate immune response to infection, in particular the production of IFN. For example the *UL83* gene product pp65 [24,25] and the IE86 protein (IE2) of HCMV and the Ie1 protein of MCMV have been reported to moderate production of pro-inflammatory cytokines [26,27]. Human CMV has also been shown to disrupt functionality of the interferon stimulated gene factor 3 (ISGF3), reducing IFNα production [28] and very recently the early gene UL26 has also been described to antagonise NFκB activation [29].

Two well-characterised inhibitors of innate immune signalling in murine CMV (MCMV) infection are the proteins M27 and M45. M27 and its HCMV homologue UL27 are efficient inhibitors of Type I and Type II IFN signalling through interaction and degradation of STAT2 and interference with tyrosine phosphorylation [30–32], therefore interfering with downstream autocrine and paracrine effects of TLR activation with the exception of plasmocytoid dendritic cells [33]. On the other hand, expression of M45 during the early phase of the infection cycle has been demonstrated to block NFκB activation, therefore interfering directly with PRR signalling pathways [34]. The mechanism of action for M45 is based on interaction with RIP1/3 and NEMO, proteins involved in the signalling cascade controlling the degradation of the inhibitor of NFκB, Ikbα [34,35]. However, *de novo* expression of both inhibitors M27 [30–32] and M45 [36] is necessary for their inhibitory activity and takes place during the early phase of the infection cycle. Of these proteins only M45 has been detected in virions [37]. Recently and unexpectedly the viral particle associated M45 protein has been shown to promote the activation of NFκB in fibroblasts during the immediate early (IE) phase of infection [36]. However, the functional relevance of this to infection is not clear at present. This poses the question if other mechanisms are in place to ensure sufficient viral gene expression despite the activation of anti-viral signalling events during the IE-phase of the CMV transcription-replication cycle.

IE-gene expression is under control of a potent enhancer that plays a critical role in determining success of a productive CMV infection [38–42]. *In vivo* the loss of the complete enhancer results in greater than a 3-log reduction in viral load and fails in exponential growth [31, 34]. Indeed, the human CMV genome has been long established to contain one of the strongest known enhancers as part of its major immediate early promoter (MIEP) with a 650 bp core that binds multiple transcription factors and which governs expression of the viral IE-genes [43–45]. While this region is functionally present in all CMV genomes the enhancer sequences are not conserved but instead share many of the same regulatory TF binding elements [41]. In particular all CMV enhancer regions contain a large number of highly redundant signal-regulated transcription factor binding sites, such as those interacting with NFκB, AP1 and CREB/ATF, factors that can be also activated by the TLR signalling pathways, [5,41,46,47]. This overlap combined with the combinatorial flexibility of the enhancer TF interactions indicates a potential for CMV to utilise the activation of anti-viral signalling pathways in the host cell. It has been reported that TLR9 stimulation plays both positive and negative roles in HCMV infection [48] and that TLR4 and TLR9 activation can increase gene expression from a human CMV enhancer expression plasmid [46]. It is thus conceivable that the CMV enhancer might advantageously co-opt the triggered TLR-signalling pathway and therefore efficiently initiate its transcription-replication-cycle before the host-cell could produce any anti-viral effector molecules [47]. The basis of this concept has been discussed before [49] however, previous models have focused mainly on the role of NFκB, placing hijacking of NFκB signalling at the centre of the co-opting strategy. Notably, for both HCMV and MCMV it has been shown that NFκB is not essential for wild-type virus to drive its gene expression and only becomes crucial when other TF binding sites are impaired [50,51]. This poses the question whether the CMV viral enhancer has evolved a functional role in effectively co-opting multiple redundant immune signal-regulated TFs for initiating a productive transcription-replication cycle.

Hence, the underlying hypothesis for the present study is that inflammatory signalling at immediate-early times may promote viral infection through viral enhancer sequences. We report our first experimental tests to refute this hypothesis by systematically investigating the requirements and mechanisms for innate immune regulation of the CMV enhancer, in particular upon infection of macrophages and upon *in vivo* infection. We use a combination of RNAi library screens with host and viral genetics to delineate the TF network controlling the enhancer. Our findings reveal an integrated inflammatory TF-network consisting of IRF5, SP1, RXR and NFκB pathways with signal activation strongly dependent on MyD88 that is delimited by a specific temporal window for activation. These results support the hypothesis and further advance the concept of viral enhancer mimicry of innate immune promoters as an immune evasion strategy.

## Results

### Host innate immune genes and viral IE-gene expression have comparable expression kinetics and respond to common inflammatory activators

**Fig 1:**
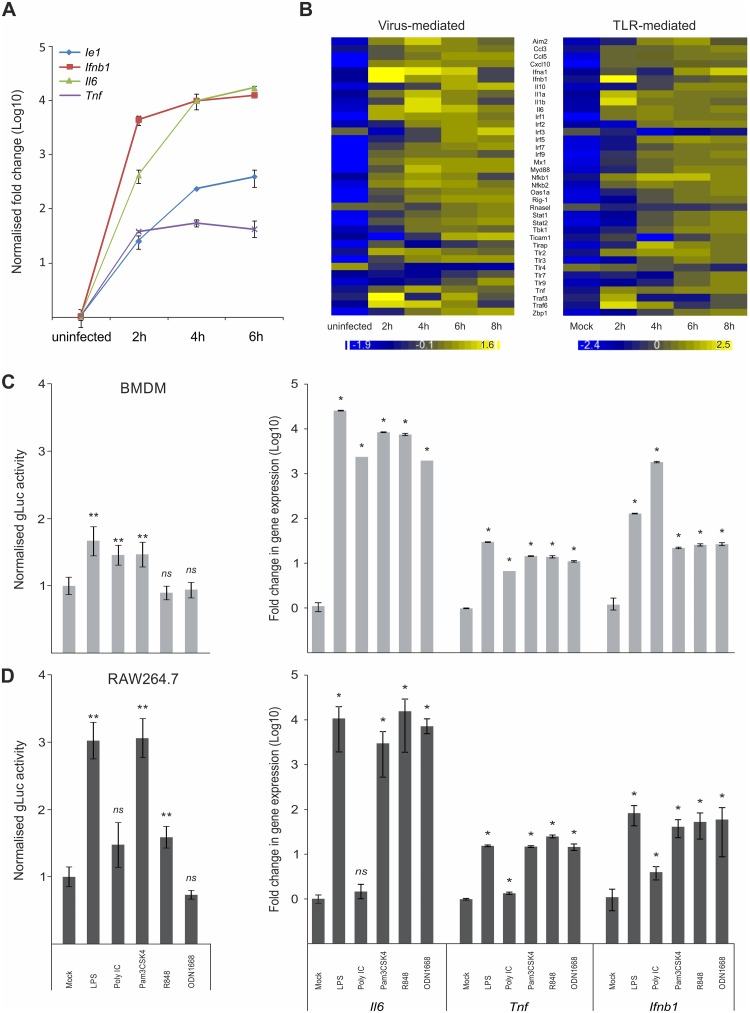
Host innate-immune response genes and viral IE-gene expression have comparable kinetics and are activated by common factors. A) Induction of gene expression for the host *Tnf*, *Ifnb1* an *IL6* genes and the viral *Ie1* gene were measured by qPCR (n = 4) after infection with MCMV. *IL6* and *Ie1* were not expressed in uninfected sample, so Ct of 36 was set as arbitrary comparison point. B) Heat maps of Microarray data for representative innate immune genes after infection with MCMV (left panel) or stimulation with Poly IC (right panel) showing comparable kinetics of gene induction after stimulation for most of the genes. Gene expression was normalised per gene to average expression levels and colour scale indicates fold change over average expression. C) Left panel: BMDMs (n = 50) were incubated with TLR-agonists for TLR4 (LPS), TLR3 (Poly IC), TLR2 (Pam3CSK4), TLR7 (R848) and TLR9 (ODN1668) for 15 min prior to infection with MCMV-gLuc to stimulate TLR signalling (for x-axis categories see Fig 1D). IE-gene expression was measured at 2 hpi by determining gLuc activity. Asterisks depict statistical significant changes identified by Wilcoxon two-sample test (* = p-value ≤ 0.05, ** = p-values ≤ 0.01, ns = not significant). Right panel: Induction of *Il6*, *Tnf* and *Ifnb1* expression in BMDMs after stimulation (4h) with the TLR-agonists (for x-axis categories see Fig 1D) used in this study with average fold changes over mock (n = 4) measured by relative qPCR. Asterisks depict statistical significant changes identified by Wilcoxon two-sample test (* = p-value ≤ 0.05). D) Left panel: Raw264.7 (n = 18) were incubated with TLR agonists and infected with MCMV-gLuc as described for Fig 1C. IE-gene expression was measured at 2 hpi by determining gLuc activity. Asterisks depict statistical significant changes identified by Welch two-sample t-test (* = p-value ≤ 0.05, ** = p-values ≤ 0.01, ns = not significant). Right panel: Induction of host cytokines *Il6*, *Tnf* and *Ifnb1* in RAW264.7 (n = 4) cells was measured as described for Fig 1C. Asterisks depict statistical significant changes identified by Wilcoxon two-sample test (* = p-value ≤ 0.05, ns = not significant).

We first sought to examine whether viral IE-genes show similar expression kinetics to host innate immune genes upon infection of macrophages and test if they react to common stimuli. For these experiments we compared the expression kinetics of the host innate immune genes *Ifnb1*, *Il6* and *Tnf* with the viral major IE-gene *M123* (*Ie1*) by relative qPCR. As expected infection of bone marrow derived macrophage (BMDM) cells from C57/BL6 mice with MCMV triggered expression of the host pro-inflammatory cytokines IFNβ, IL6 and TNF (Fig. 1A). Notably, the overall kinetics of the viral *Ie1* gene and the host factors are similar, with a rapid induction of gene expression within the first 2 h post infection (hpi). To more extensively examine the induction of host innate immune genes we performed a microarray clustering analysis of expression levels in BMDMs after MCMV infection (Fig. 1B). For this study we used a set of well-known innate immune genes and TLR signalling components. This analysis revealed genes with expression profiles similar to the *Ie1* expression profile shown in Fig. 1A, with a rapid induction within 2–4 hpi (Fig. 1B, left panel). Exceptions of this pattern were *Il10*, *Tlr4*, *Tlr7* and *Tlr9*. *Il10*, *Tlr7* and 9 were induced with delayed kinetics and reaching peak expression levels by 6 h or later. In contrast, *Tlr4* seemed to have a high level of steady state expression in the mock sample and was down regulated after infection. To determine whether these changes in gene expression can be, at least partially, triggered by TLR activation alone, we further analysed the same set of genes in BMDMs stimulated with Poly IC, which activates TLR3 signalling (Fig. 1B, right panel). The stimulation with Poly IC recapitulates the observed pattern triggered by CMV infection with the exception of TLR4 expression. The down regulation of TLR4 observed in the infected sample was strongly delayed after Poly IC challenge. This might reflect the synergistic effect of parallel activation of several PAMPs by the infection process, which could explain the more pronounced down regulation in the infected versus the single stimulus by Poly IC.

Since HCMV-enhancer-driven reporter plasmids have been shown to positively respond to LPS and CpG [46], we tested if the viral IE-gene expression responds to stimulation of TLR signalling in the context of the viral infection. For these experiments RAW264.7 and primary BMDMs were pre-stimulated for 15 min with ligands for TLR4, TLR3, TLR2, TLR7 and TLR9 to stimulate the TFs activated by TLR signalling. These cells were subsequently infected with a gaussia luciferase [52,53] reporter virus (MCMV-gLuc) to quantitatively measure in the context of infection the activity of enhancer. This recombinant virus (S1 Fig for structure and mutagenesis strategy) has had the dispensable *m128* (*Ie2*) gene [54–56] replaced by a reporter cassette expressing a *gaussia luciferase* (*gluc*) reporter under direct control of the murine CMV enhancer. Levels and kinetics of *gluc* and *Ie1* expression in this reporter mutant are comparable as demonstrated by qPCR measurement (S2 Fig). Subsequent to TLR stimulation the cells were infected and 2 hpi the activity of the secreted gLuc reporter was measured in the cell culture supernatant (uninfected background for BMDMs was determined as 370.28 RLU with SEM = 4.81, n = 35 independent biological experiments; uninfected background for RAW264.7 was 82 RLU with SEM = 1.44, n = 32). As shown in Fig. 1C (left panel) and Fig. 1D (left panel), both cell systems showed a significant increase in reporter gene expression for specific TLR ligands (pre-normalised average of RLU for mock in BMDMs = 3.23x10^4^ RLU with min = 1.26x10^3^ and max = 1.69x10^5^, n = 50; not normalised average mock for RAW264.7 = 4.04x102 RLU with min = 9.6x10^1^ and max = 9.39x10^2^ RLU, n = 18). Notably, there are differences between the monocytic cell line RAW264.7 and the primary BMDMs in the measured levels of gene expression, for TLR4, TLR2 and TLR7 ligands LPS, Pam3CSK4 and R848, respectively. In these cases the RAW264.7 cells show a stronger response to the respective ligands, which is most likely related to differences in expression levels of their respective TLR repertoire. We therefore compared the expression levels of selected TLRs between BMDMs and RAW264.7 cells and found all of the assessed TLRs had increased expression levels in the RAW264.7 cells (S3 Fig). We furthermore could find no significant changes in uptake of viral genomes after TLR ligand treatment in BMDMs (S4 A and B Fig), indicating that the observed increase in gLuc activity is due to increased gene expression. To check if the used TLR ligands are biologically active and can induce host innate immune gene expression, we measured induction of *Ifnb1*, *Il6* and *Tnf* in primary BMDMs and RAW264.7 by the same TLR ligands (Fig. 1C and Fig. 1D, right panels). Notably, Poly IC was ineffective in activating gene expression in RAW264.7 cells in contrast to BMDMs. This might be due to differences in uptake of Poly IC since TLR3 is mainly localised in endosomes and its subcellular location is cell type dependent [57–60]. Taken together these data clearly show that inflammatory stimuli that induce expression of innate immune genes also enhance viral IE-gene expression in the analysed cell systems.

Altogether we conclude that the CMV enhancer is activated with similar kinetics to innate immune genes in the context of infection and is positively responsive to inflammatory TLR-signalling.

### A temporal gate for TLR-triggered signalling to boost viral enhancer activity and replication

**Fig 2:**
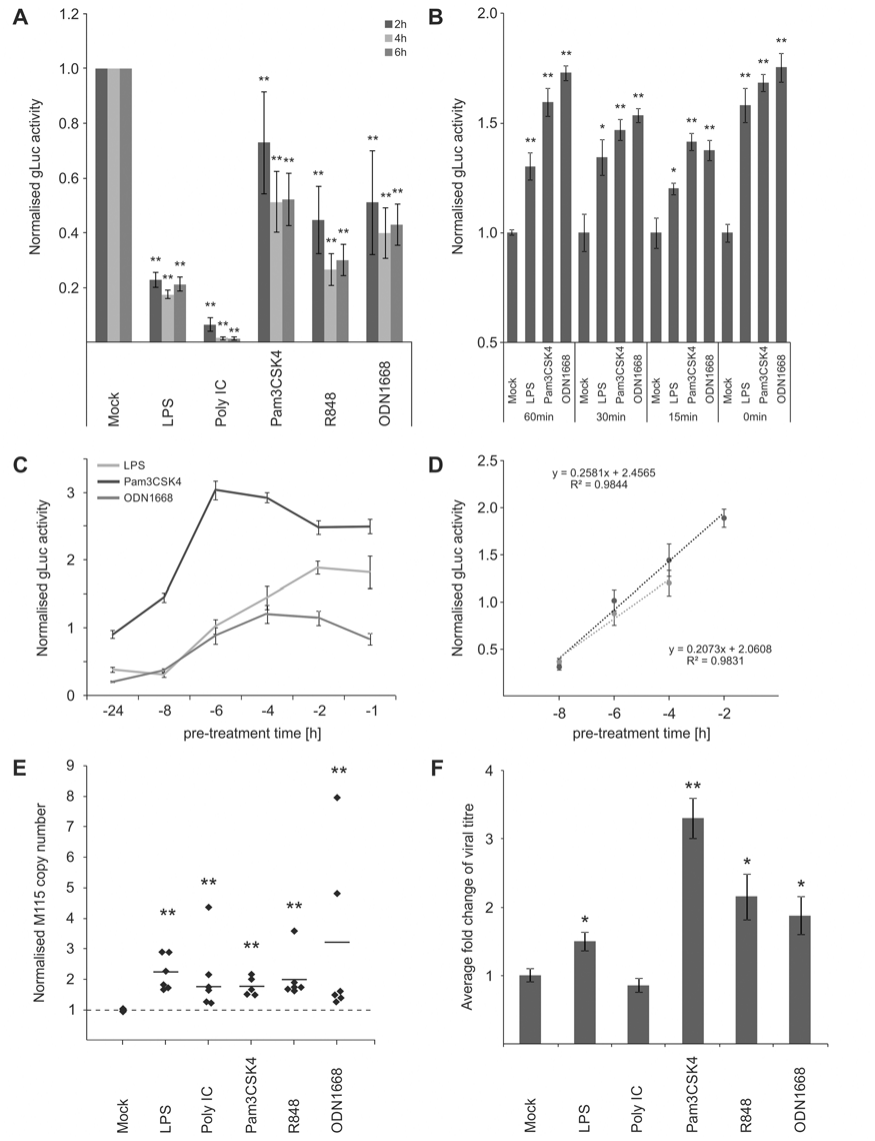
IE temporal gate for TLR-triggered signalling to boost IE-gene expression and viral replication. A) Inhibitory effects of long-term pre-treatment with TLR ligands. BMDMs were incubated for 24 h with indicated TLR ligands and subsequently infected (MCMV-gLuc). Reporter activity was measured at indicated time point p.i. Bars represent mean of normalised activity from n = 64 with error bars showing SEM and asterisks indicating statistical significant changes identified by Wilcoxon two-sample test (* = p-value ≤ 0.05, ** = p-values ≤ 0.01, ns = not significant). B) and C) IE-Time kinetic experiments of TLR-agonist pre-treatment and its effect on MIEP activity. BMDMs were pre-treated for indicated time points and infected with MCMV-gLuc (MOI 0.2). Reporter activity in culture SN was measured at 3 hpi (B, n = 6) or 4 hpi (C, n = 8). Averages of cultures are shown with SEM, normalised to mock activity and asterisks indicate statistical significant changes identified by Welch two-sample t-test (* = p-value ≤ 0.05, ** = p-values ≤ 0.01). D) Linear phases of time-effect curves for TLR4 (black circles) and TLR9 (grey circles) agonists were estimated from plots in Fig 2C. Linear trend-lines were fitted to the respective data points (average with SEM) and were used to determine ET50 values (TLR4 = -5.25 h; TLR9 = -6.15 h). E) Quantification of viral genomes in infected BMDMs (MCMV-gLuc) shows increased numbers of genomes for all used TLR-agonists. Genome copy numbers were absolutely quantified, corrected by *Gapdh* as cellular control and then normalised to copy numbers from mock-treated samples. Dotted line indicates level in mock samples and thick solid black lines mark the average of the treatment groups (n = 6). Asterisks indicate statistical significant changes over the mock group identified by Wilcoxon two-sample test (** = p-value≤0.01). F) Measurement of viral replication in BMDMs by plaque assay (MCMV-gLuc). Supernatant from n = 6 infected BMDM cultures were harvested at day 3 p.i. and assayed for infectious viral particles on *Stat1* ^-/-^ MEFs. Plaque numbers were normalised to average of mock samples (2.8x10^3^ PFU/ml) and asterisks indicate statistical significant changes over mock identified by Welch two-sample t-test (* = p-value≤0.05; ** = p-value≤0.01).

Our results so far indicate that stimulation of TLR signalling close to the time point of infection is sufficient to increase viral enhancer activity. While this concurs with work that demonstrated a stimulation of human CMV reporter-plasmids after LPS/CpG treatment, it is in contrast to the well-established observation that activation of TLR signalling is necessary for resistance to pathogens such as CMV [15]. As shown in Fig. 2A, long term pre-treatment (24 h) of BMDMs with all TLR ligands used in this study significantly inhibits MCMV as expected. The inhibitory effects of the TLR-ligands are most likely due to the induced expression of anti-viral effectors such as IFNβ (compare with Fig. 1C and 1D) that subsequently establish an anti-viral state through autocrine and paracrine effects [61–63].

For the anti-viral effector molecule IFNγ we have shown previously [64], that it has a half maximum pre-treatment time (ET_50_) of 1.5 h to impart 50% inhibition of CMV enhancer activity in BMDMs. Since production of IFNs in naive cells needs to be induced first, this indicated that there should be a lag in the system between first contact with the virus and production of IFNs. In agreement, we have previously found that upon infection of BMDMs, IFNβ secreted protein levels peak by 6 hpi [64]. This lag predicts a possible temporal gate that would be open for a period no more than 7 hours for MCMV to establish infection before anti-viral effectors fully inhibit the virus.

Therefore we sought to determine if the anti- and pro-viral effects we observed are dependent on a time window and if so to define the boundaries of this potential temporal gate by comparing different pre-treatment times ranging from 0–24 h. When we tested pre-treatment times with ligands for TLR2, TLR4 and TLR9 ranging from parallel treatment (0 min) to 60 min pre-treatment, we found that all conditions were pro-viral, showing that there seems to be no measurable lower limit for the temporal gate (Fig. 2B). To establish the upper limit of the temporal gate we tested pre-treatment times ranging from 1–24 h. Fig. 2C shows that the effects of TLR ligands for TLR2, TLR4 and TLR9 changed over time from anti-viral to pro-viral with decreasing pre-treatment times. Matching the observations shown in Fig. 2A, the TLR ligands showed different levels of anti-viral activity with 24 h pre-treatment, with TLR2 showing only weak anti-viral activity and becoming pro-viral from 6 h or less. The more highly potent anti-viral states induced by TLR4 and TLR9 agonists, required shorter pre-treatment times to establish resistance to infection. To more precisely quantify the temporal window we determined the half maximal time for pro-viral enhancer stimulation by computing the ET_50_ for the TLR agonists. We fitted a regression function to the linear phase of the response (Fig. 2D) and estimated the ET_50_ values as -5 h 15 min for TLR4 and -6 h 9 min for TLR9, respectively.

Therefore, these experiments revealed the existence of a temporal gate in which CMV IE-gene expression can co-opt TLR signalling to its advantage, within the first 6 h of infection.

We next sought to investigate if the observed boost in enhancer activity conveyed a benefit for viral production. We assessed effects of TLR signalling on viral replication by quantifying replication of the viral genome and the production of infectious particles. To measure viral genome replication we used absolute quantification of viral genome copies by qPCR in infected BMDMs at 24 hpi, a time point at which the first round of replication is completed [65]. Detection of the host gene *Gapdh* was used to correct for potential variation in the amount of input material and values were furthermore normalised to the copy numbers of the mock sample (average genome copy number without normalisation was 1.73x10^5^ with min = 3.28x10^3^ copies and max = 2.5x10^5^ copies). Fig. 2E shows that 15 min pre-treatment with all tested TLR ligands significantly increased viral genome replication compared to the mock control. We then tested the impact of TLR activation on the production of infectious viral particles by standard plaque assay. To reduce interference of IFNs produced by the infected experimental BMDM cultures and to increase sensitivity we used *Stat1* ^-/-^ MEFs for the plaque assay. As shown in Fig. 2F all treatments, except for Poly IC, significantly increased the production of viral particles in the culture at 3 days post infection. While quantitative variation in IE gene expression is observed for the different ligands, the levels of boosted IE-gene expression and viral genome replication do not completely reflect the detected increase in viral particle production in the plaque assay. Although we do not fully understand this variation it is most likely due to the downstream anti-viral factors and their effects elicited by the respective TLR signalling pathways (compare to Fig. 1 and Fig. 2A).

Nevertheless these data demonstrate that TLR activation can boost at IE-times of infection both viral gene expression and subsequent replication and supports the notion that CMV enhancer might co-opt activity of innate immune signalling to its own advantage.

### A direct effect of TLR-signalling on CMV enhancer activity

**Fig 3:**
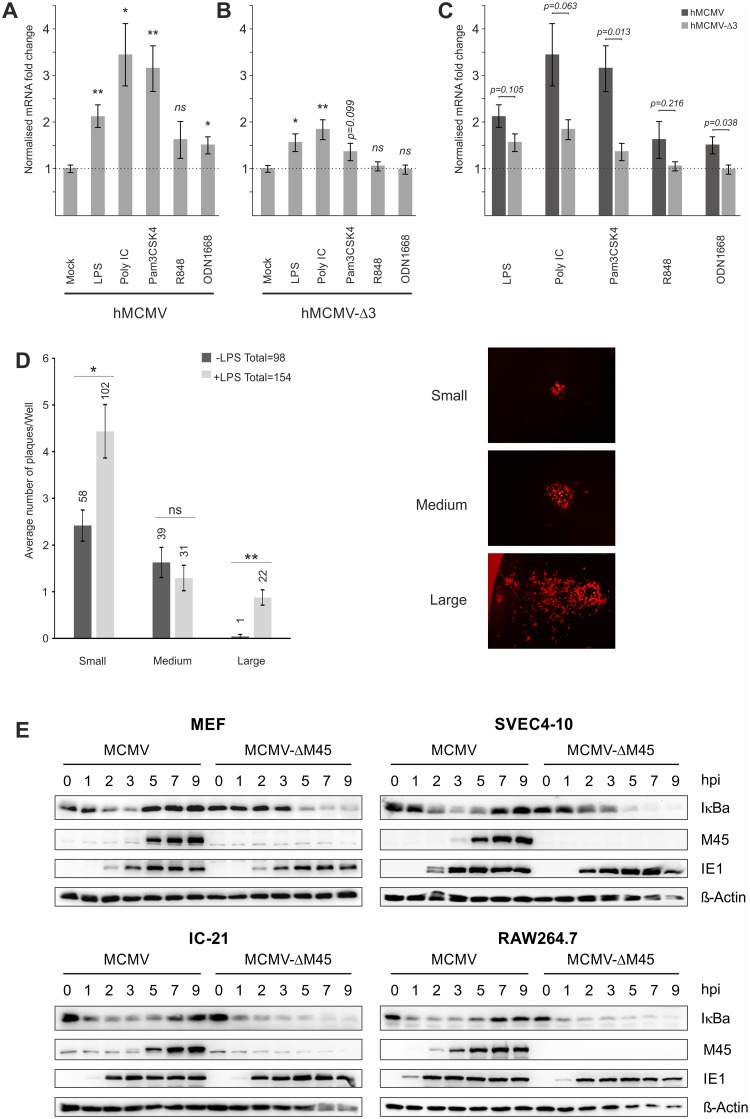
Effects of TLR-agonists go beyond NFκB activity and are independent of tegument proteins. Comparison of 6 biological replicates measuring *Ie1*-gene expression of chimaeric MCMV recombinant viruses carrying either a human CMV enhancer (hMCMV) (A) or a human CMV-NFκB/AP1/ATF-motif-disrupted-enhancer (hMCMV-Δ3) (B) after stimulation with indicated TLR-agonists in BMDMs by qPCR. Expression was normalised to *Gapdh* and is shown relative to mock treated samples and asterisks indicate statistical significant changes over mock for each group as identified by Welch two-sample t-test (* = p-value≤0.05; ** = p-value≤0.01, ns = not significant). C) Statistical comparison (two-sample Welch’s t-test) of differences in fold changes between hMCMV and hMCMV-Δ3 viruses for each TLR-agonist (n = 6). D) TLR stimulation acts independent of delivered tegument proteins. Primary MEF cultures were transfected with MCMV-gLUC DNA (16 h) and subsequently either mock (n = 24) or LPS (n = 24) stimulated. At 6 days post stimulus viral plaque production was quantified and plaques were classified into small, medium and large categories (representative plaques for the categories are shown). Bars depict average plaque numbers (indicated above each bar) with SEM. Statistical significance of observed differences between treatments is indicated by asterisks (Mann Whitney U Test, * = p-value≤0.05; ** = p-value≤0.01; ns = not significant). E) In monocytes NFκB activation and IE1 protein levels are not dependent on M45. Representative western blots detecting proteins IκBa, M45, IE1 and b-Actin in primary MEFs, SVEC4-10 endothelial cells, IC-21 and RAW264.7 macrophage cells infected with either MCMV or MCMV-ΔM45 virus (MOI of 10 TCID_50_/cell). Cells were lysed at the indicated times post-infection, and protein levels determined by immunoblotting.

Comparison of TFs known to bind the HCMV enhancer with TFs activated by TLR signalling show that several factors are shared, including NFκB, AP1 and ATF [41,66–68]. To analyse the importance of these TFs in our system we used a set of chimaeric viral recombinants in which the human CMV wild-type and mutant enhancers replace the native murine CMV enhancer [38,69]. We analysed the effects of TLR activation on *Ie1* gene expression in the chimaeric virus carrying the wild-type human CMV enhancer (hMCMV) and compared this with a triple knockout mutant (hMCMV-Δ3) in which all enhancer binding motifs for NFκB, AP1 and ATF have been rendered non-functional by point mutations. As can be seen in Fig. 3 the disruption of the binding motifs significantly reduced the expression of *Ie1* after TLR stimulation, demonstrating that the observed effects are in part due to direct activation by NFκB, AP1 and/or ATF. However, we note that the mutation of all NFκB, AP1 and ATF binding sites could not completely abolish the boost of viral *Ie1* gene expression by TLR activation. Stimulation of TLR4 (p-value≤0.05), TLR3 (p-value≤0.01) and, with a trend, TLR2 (p-value = 0.099) was still able to boost viral gene expression. This result indicates that additional host factors must also be involved in the stimulation of CMV enhancer activity by TLR signalling.

It is possible that a viral tegument protein rather than indirect TLR signalling could lead to activation of TFs. A very recent publication showed that the viral protein M45, which is delivered into cells initially by viral particles, is a potent activator of NFκB signalling at IE times despite its role as an inhibitor of NFκB at early and late times of infection [36]. However, these studies were limited to fibroblasts and found no necessity for M45 to activate viral IE-gene expression. To assess whether M45 or other tegument proteins are involved in the observed TLR-mediated effects on IE-gene expression, we first examined the effect of TLR stimulation on genomic MCMV-gLuc DNA transfected into primary MEFs. After the transfection cells were visually checked for successful transfection by assessing if individual fluorescent cells were present in the cultures. Subsequently, half of the transfected cultures were treated with LPS to trigger TLR signalling. Over the first 8h we checked repeatedly for changes in expression of the reporter gene *gLuc* and found that from 4h post treatment a shift in in a number of independent LPS treated cultures to higher reporter expression was detectable. To determine if this initial boost of gene expression translated into increased viral replication, we assessed the number of plaques and the size of plaques at 6 days post treatment (Fig. 3D). This analysis showed that the group of LPS treated cultures produced more viral plaques in total and had statistically significant higher numbers of plaques in the small and large categories. It is noteworthy that large plaques were mainly present in the LPS treated group. This experiment demonstrates that an initial boost through TLR stimulation can translate into an enhancement of the transcription-replication cycle from viral genomic DNA. This observation further supports the notion that the observed effects are not absolutely dependent on viral tegument proteins.

Since it has been recently demonstrated that in NIH3T3 fibroblasts the activation of NFκB immediately after infection is strictly dependent on M45 [36], we next assessed the role of this tegument protein in the macrophage system using a ΔM45 recombinant virus. A comparison of protein levels in MEFs infected with wild type and mutant virus confirmed the phenotype published for NIH3T3 cells. In MCMV infected MEFs a rapid degradation of the inhibitor of NFκB (IκBa) could be observed that was blocked at later times (≥5 hpi) when *de novo* synthesised M45 became visible (Fig. 3E, top left panel). In MEFs infected with the MCMV-ΔM45 virus the degradation of IκBa only became detectable at 5 hpi. In contrast to this, rapid degradation of IκBa was observed in the SVEC4-10 endothelial cell line and in the macrophage cell lines IC-21 and RAW264.7 after infection with both, the MCMV and MCMV-ΔM45 viruses (Fig. 3E, top right and lower panels). This demonstrates that, in contrast to fibroblast cells, NFκB activation at IE-times in infected endothelial and macrophages is not dependent on M45. Overall, the loss of M45 did not overtly impact on IE1 protein levels in any of the tested systems. In separate experiments we also find the M45 MCMV mutant was still responsive to TLR activation in macrophages by stimulation with TLR2 agonist (S5 B Fig).

We conclude from these experiments a direct involvement of the enhancer elements in mediating responsiveness to TLR-signalling and while not mutually exclusive from the possible contribution of viral particle associated proteins, is shown to be independent of the virion-delivered M45 protein.

### Loss-of-function screen for enhancer activity identifies an integrative network of immune activated transcription factors linking retinoid receptor activation and TLR stimulation

**Fig 4:**
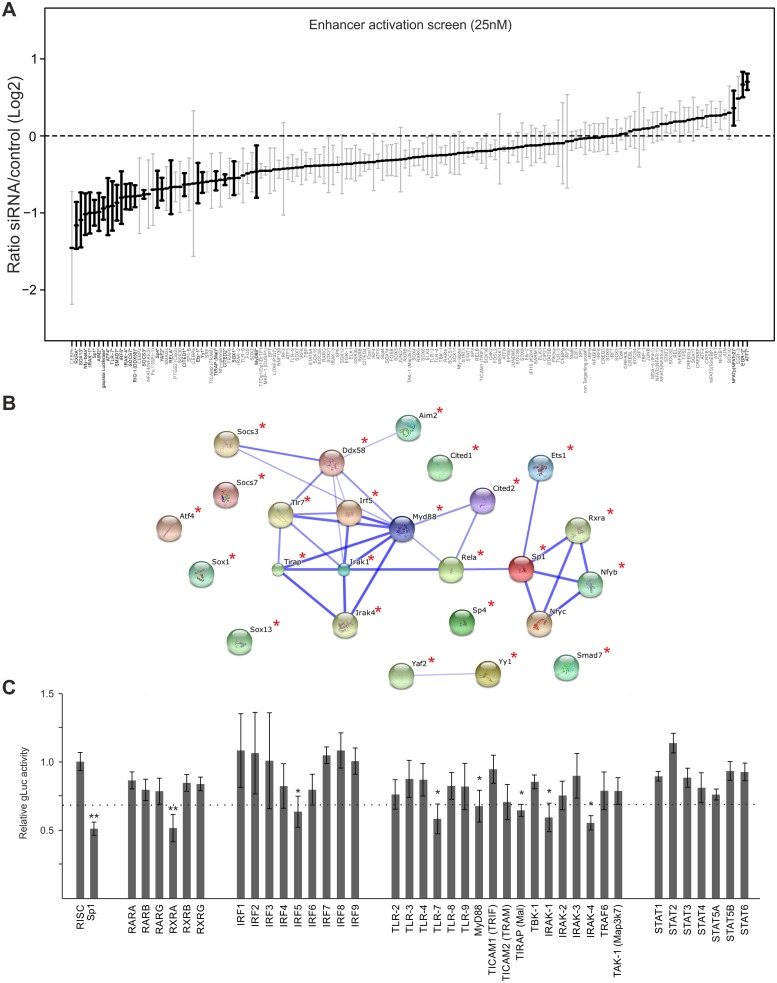
Small interfering RNA screens identifies TLR-signalling network. A) GLuc activity data from all 25nM siRNA screens (n = 4). Reporter activity shown is normalised to RISCfree control siRNA. Black circles and bold gene name indicate statistically significant effects for corresponding gene knockdown (Wilcoxon test; p-value≤0.05). B) STRING network of the candidate genes that showed >75% maximal knockdown effect compared to controls in all 25nM siRNA screens (n = 4). Asterisks indicate statistical significant knockdown (p-value≤0.05). Corresponding data is shown in S2 Table. C) GLuc activity data for the RXR-network, the IRF family and the TLR network, from all 25nM siRNA screens (n = 4). Reporter activity shown is normalised to RISCfree control siRNA. *Sp1* siRNA knockdown show maximally achieved inhibition and asterisks indicate statistical significant knockdowns (Wilcoxon test; * = p-value≤0.05; ** = p-value≤0.01) and dotted line depicts 75% maximal knockdown cut-off used to identify candidates for STRING network.

To explore more fully the TF network required for enhancer activation, we used a library of small interfering RNAs (siRNAs) for targeted knock-down of immune signalling components and TFs that are either known to bind the CMV enhancer or to be activated by TLR signalling and a range of positive and negative control genes (targeting in total 149 host factors, complete list of targets are shown in S1 Table). We transiently transfected primary MEFs with targeted SMARTpools (Dharmacon, Lifetechnologies) and subsequently infected them with the gLuc-reporter virus (MCMV-gLuc) to monitor exclusively enhancer activity at 6 hpi (25nM siRNA screens). Fig. 4A shows the summarised results of 4 independent, normalised gLuc screens that passed the quality control filter (detectable expression of the reporter in the mock transfected samples and knock-down by several positive control siRNAs, e.g. targeting either the reporter *gLuc* or TFs known to be important for CMV enhancer activation, e.g. *Sp1* [68,70]). Due to a high level of redundancy in the CMV enhancer overall inhibitory effects of the knockdowns rarely reach more than two-fold, even with the positive controls. We therefore ranked the siRNA knockdowns relative to the maximal achieved knockdown by the *gLuc* targeting positive control siRNA (Ranked list of all genes see S1 Table, for corresponding knock-down data see S2 Table). For analysis we set cut-offs representing different ranks of our 149 targeted host factors. We used three levels of stringency to sort our target list; the high stringency group (>75% of maximal knock-down effect) consisted of the top 25 candidate genes, the medium stringency group (>50%) included the top 63 genes and the low stringency group (>25%) included the top 101 gene candidates.

To investigate whether the hits from the screen were dispersed over a range of different interaction networks or were limited to a discrete network of biochemical/molecular interactions we undertook a network analysis of the high stringency group of 25 candidates with the STRING web tool. This approach determines edge connectivity of the hits based on known and predicted molecular interactions [71]. The results of this analysis shown in Fig. 4B, reveal that most of the target genes of the >75%-group could be mapped to a principal network with the TLR-adaptor protein MyD88 at its centre (confidence of interaction is indicated by thickness of connecting edges, asterisks next to network node indicates statistical significant knock-down) and a link to an RXR network. We found that a substantial part of our statistically significant hits could be mapped to the TLR immune response pathway (top hit in GO term enrichment test was “activation of innate immune response” with *p-value*≤7.609x10^-9^) with associated innate immune factors, such as TLR7, IRAK1/4, MyD88, IRF5 and also RIG-I (Ddx58), and AIM2. This functional network was connected to the NFκB subunit RelA as was to be expected but also included the TFs, SP1, ETS1, Nfyb, Nfyc and RXRA. The presence of RXRA, ETS1 and SP1 in the list of significant hits affecting viral enhancer activity was not surprising, since interactions with the CMV enhancer for SP1 [70], the ETS-family (ELK1 [72], ETS-2 [73]) and RXRA [41,74–76] have been described in the literature. In support, Fig. 4C shows the normalised average gLuc activity of all members of the RXR network, the IRF family and the TLR signalling components for comparison. Notably, the factors IRF5, AIM2, RIG-I, Nfyb and Nfyc have not been previously implicated in mediating activation of CMV gene expression.

An analysis of the medium and low stringency factors with the topology inferred by the STRING software tool showed a highly integrated network, comprising TLR signalling components linked to the RXR network (see S6 and S7 Figs).

### Statistical meta-analysis of an extended series of independent screens for viral growth and enhancer activity identifies divergent and a convergent NFκB-SP1-RXR-IRF transcription factor axis

**Fig 5:**
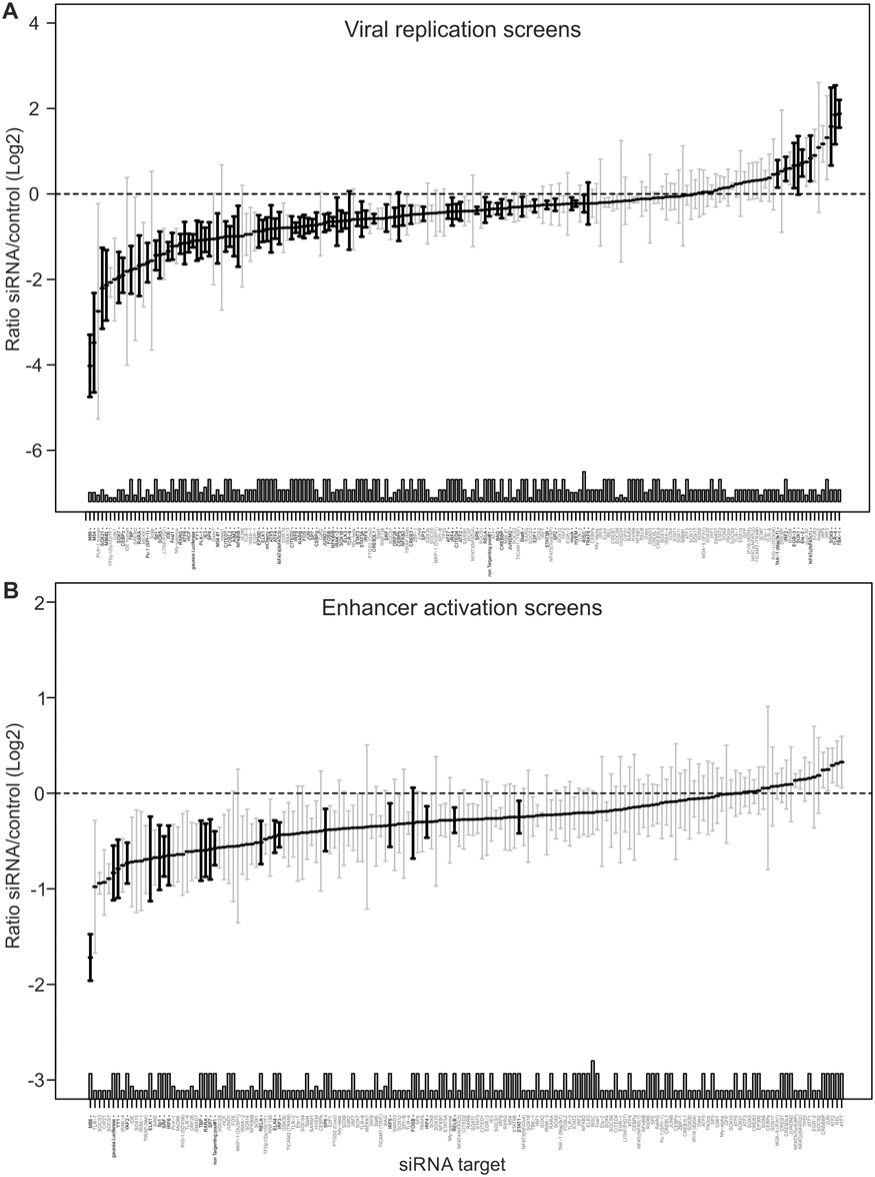
Summary of all Loss-of-function siRNA screening data. Ordered lists with all values for siRNA targets on x-axis available in S3 Table. A) Viral replication: Median (and bootstrapped standard error of median) of fold change (y-axis) between siRNA and infected controls, measured by GFP activity across all screens (MCMV-GFP). The numbers of independent screens for a particular siRNA is available in bars (on x-axis) and are n = 4, 6, 8, 10, 12, 18 or 24. Data points highlighted in bold are significantly different (by Wilcoxon Signed Rank test) from a zero fold change, i.e. infected controls. B) IE-gene expression: Median (and bootstrapped standard error of median) of fold change (y-axis) between siRNA and infected controls, measured by gLuc activity (MCMV-GFP) across all screens (n is 3, 4, 7 or 10), symbols as described in (A).

We next sought to assess whether transcription factor requirements are convergent or divergent for viral growth and enhancer activity. Due to the inherent redundancy of TF requirement for enhancer activity we, therefore, conducted a large number (up to n = 24) of systematic independent siRNA screens for the 149 TFs. For these screens we used a GFP-expressing reporter virus (MCMV-GFP) to monitor viral replication at 72 hpi and MCMV-gLuc to monitor enhancer activity at 6 hpi. By applying a robust statistical meta-analysis we aimed to increase the statistical power and identify the most consistent siRNA effects on the results of all screens over all experimental conditions. Fig. 5 shows the medians of each siRNA over all screens from all experimental conditions with those highlighted in bold being significantly different from the infected controls (number of screens per siRNA up to n = 24, see figure legend). The upper panel of Fig. 5 shows replication efficiency at 72 hpi, while the lower panel shows enhancer activity at 6 hpi (see also S3 Table). This analysis allowed for statistically stringent assessment of viral replication and enhancer activation screens and revealed, as expected from the literature, components of the TLR signalling pathway, such as TAK1, TBK1 and IRF2 that have a statistically significant anti-viral effect when measuring replication.

In contrast and in concordance with the temporal gate model for immune activation we also find many of the TLR signalling components, such as TLR9, IRAK2, IRAK4 and IRF3, IRF4, IRF5 and IRF6 to have a pro-viral effect shown in the lower panel of Fig. 5. For significant hits and overlap between the two screens see Table 1. Assessing the overlap between the two approaches, we find several genes that significantly reduced enhancer activation as well as viral replication, namely ELK1, FOSB, IRF4, IRF5, IRF6, RELA, RXRA, Sp1, SP6, SP7, SRF and YY1. Notably, IRF5, RELA, RXRA, SP1 and YY1 were also part of the network identified in the initial enhancer activation screen (Fig. 4). Taken together these targets indicate that discrete innate immune signalling plays an important role in activating the viral enhancer.

**Table 1:** Comparison of significant siRNA hits for viral replication and enhancer activation from statistical meta-analysis.

Viral replication		Viral replication		Enhancer activation	
siRNA target	effect of knock-down on virus	siRNA target	effect of knock-down on virus	siRNA target	effect of knock-down on virus
AIM2	-	RARB	-	ELK1*	-
ATF2	-	RELA*	-	ELK4	-
ATF4	-	RXRA*	-	FOSB*	-
ATF5	-	RXRG	-	IRF3	-
ATF6	-	SOX1	+	IRF4*	-
ATF7	-	SOX18	-	IRF5*	-
CEBPb	-	SOX21	-	IRF6*	-
CEBPg	-	SOX5	-	RELA*	-
CEBPz	-	SOX7	-	RELB	-
CITED1	-	SOX9	+	RXRA*	-
CITED2	-	Sp1*	-	Sp1*	-
CITED4	-	SP2	-	SP6*	-
CREB3	-	SP3	-	SP7*	-
CREB3L1	-	SP4	-	SRF*	-
CREBBP	-	SP6*	-	STAT1	-
EGR-3	+	SP7*	-	YAF2	-
ELK1*	-	SRF*	-	YY1*	-
ELK3	-	STAT3	-		
Ets-2	+	STAT5A	-		
FOS	-	STAT5B	-		
FOSB*	-	Stat6	-		
Fosl1	-	TAK-1	+		
FOSL2	-	TBK-1	+		
IRAK-2	-	TLR-4	+		
IRF2	+	TLR-9	-		
IRF4*	-	TRAF6	-		
IRF5*	-	YY1*	-		
IRF6*	-				
JUN	-				
JUND1	-				
JUNDM2	-				
NFAT4	-				
NFATc	+				
NFKB2	-				
NFKBIB	-				
NFkBIB	-				
Pu.1	-				

* = indicates siRNA hit overlaps between both screen types

Overall this data implies that the TLR-activated host factors NFκB (RelA), SP1, RXR and members of the IRF family play a central role in activating the enhancer in infection and that these factors may form a functional network. Thus, the TLR signalling pathway might be necessary for normal IE-gene expression levels in infections with the potential for cooperation with the retinoic-acid signalling pathway.

### Retinoic acid pre-treatment cooperatively increases the effects of TLR-ligands on viral enhancer activity

**Fig 6:**
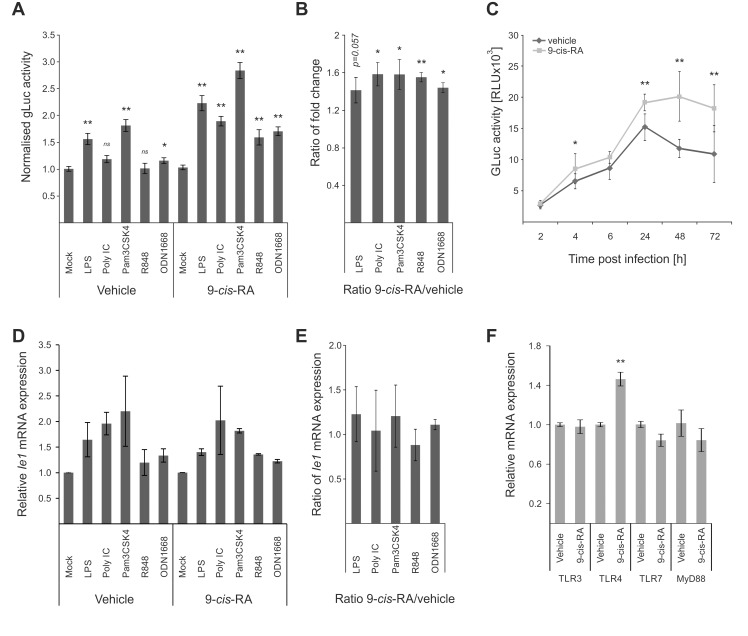
Retinoic acid pre-treatment increases effect of TLR ligands on IE-gene expression. A) Characterisation of the effects of 9*-cis* RA pre-treatment of BMDMs prior to TLR stimulation. Cells were pre-treated for 24 h before TLR stimulation and infection (MCMV-gLuc). Gluc activity was measured at 2 hpi and is shown relative to mock treated samples. Average of n = 32 with SEM is shown and asterisks indicate statistical significant changes over mock for each group (Wilcoxon test; * = p-value≤0.05; ** = p-value≤0.01, ns = not significant). B) Ratios of gLuc activities in 9*-cis* treated cultures over vehicle treated controls derived from data shown in panel A. Bars show averages of 4 independent experiments with SEM and asterisks indicate means that are statistical significant different from 1 as tested by a one-sample t-test versus a constant (* = p-value≤0.05; ** = p-value≤0.01). C) Time kinetic of gLuc activity in BMDMs after 9*-cis*-RA treatment without further TLR stimulation. BMDMs were pre-treated for 24 h with 9*-cis*-RA or vehicle and subsequently infected (MCMV-gLuc) and gLuc activity was measured at indicated time points. Data points represent averages (n = 8) with SEM. Asterisks indicate statistical significant differences between vehicle and 9*-cis*-RA treated cultures for each time point (Wilcoxon test; * = p-value≤0.05; ** = p-value≤0.01). D) Deletion of RA response elements (RARE) on the viral genome leads to a loss of the increased sensitivity for TLR stimulation triggered by 9*-cis*-RA. BMDMs were pre-treated with 9*-cis*-RA or vehicle before infection with a chimaeric hMCMV-ΔRARE mutant. At 4 hpi RNA was isolated and analysed by qPCR for *Ie1* expression (n = 2). E) Average of *Ie1* expression ratios of 9*-cis*-RA treated BMDMs over vehicle treated samples derived from data shown in panel D. F) 9*-cis*-RA treatment effects on expression on TLR signalling components. Expression of TLR 3, 4 and 7 and MyD88 were measured in RAW264.7 after 9*-cis*-RA treatment (n = 7, mean with SEM is shown). Asterisks indicate statistical differences in expression over vehicle (two sample Welch t-test; ** = p-value≤0.01).

The above-described screens identified RXRA as part of the integrated TLR-network affecting viral IE-gene expression. While retinoic acid receptors have been shown to bind to and regulate human and murine CMV enhancer activity [74,77], it is known that retinoic acid receptors can also positively influence TLR expression [78,79]. To functionally test the effects of retinoids in our system, we pre-treated BMDMs with the RAR/RXR ligand [80] *9-cis*-retinoic acid (*9-cis*-RA) for 24 h prior to TLR-pre-treatment. As shown in Fig. 6A, we find that the *9-cis*-RA pre-treatment triggers an increased sensitivity of our cells to the effects of TLR pre-treatment. The ratios of gLuc activity between *9-cis*-RA-treated and vehicle-treated samples show that RA has a broadly positive effect on the system, since all TLR treatments, independent of the ligand, showed ratios significantly larger than 1 (Fig. 6B). This suggests that the viral enhancer may also directly benefit from RA stimulation. As can be seen in Fig. 6C, we monitored gLuc expression in vehicle and *9-cis*-RA treated BMDMs over a time course and observed that the *9-cis-*RA treatment increases IE-gene expression for up to 72 hpi in absence of any prior TLR ligand treatment.

The direct effects of RA on viral gene expression are mediated through multiple high affinity retinoic acid receptor binding sites (RA Response Elements, RAREs) that have been previously characterised for both the HCMV and MCMV enhancers and shown to influence IE-gene expression [74–76]. To analyse if the *9-cis*-RA treatment has a direct effect we used a chimaeric murine CMV mutant similar to those described above, in which all RAREs in the human CMV enhancer have been disrupted by point mutations (hMCMV-ΔRARE) and measured *Ie1* expression by qPCR. We found reduced levels of *Ie1* expression with the hMCMV-ΔRARE mutant compared to the parental hMCMV virus (S8 Fig). Fig. 6D shows that disruption of all RAREs in the viral enhancer still allows TLR-ligands to stimulate IE-gene expression after pre-treatment with the vehicle or 9-*cis*-RA but the additional boost observed with the MCMV virus (Fig. 6C) is lost with the hMCMV-ΔRARE mutant infection. These findings suggest that the RAREs are not necessary for TLR activation of the enhancer but are required for cooperative activation. In agreement, the ratios of *Ie1* mRNA expression between *9-cis*-RA and vehicle treated samples were not statistically significantly >1 after infection with the mutant virus (Fig. 6E), and thus demonstrate that the RAREs in the viral enhancer contribute to the enhanced sensitivity for TLR stimulation after RA treatment. However, the involvement of the enhancer RAREs is not mutually exclusive from an additional contribution involving an increase in TLR expression triggered by RA that has been implicated in other cell systems [79]. However, in experiments that measured *Myd88* and TLR expression in BMDMs after vehicle or 9*-cis*-RA treatment (Fig. 6F) we could only find a significant change in TLR4 alone, which may explain the TLR4 synergistic effect in the presence of 9-*cis*-RA but not the general increase found for the other TLR-agonists.

Taken together we conclude that *9-cis*-RA can cooperatively enhance viral enhancer activity with TLR-signalling.

### A MyD88-dependent pathway contributes to CMV enhancer activity

**Fig 7:**
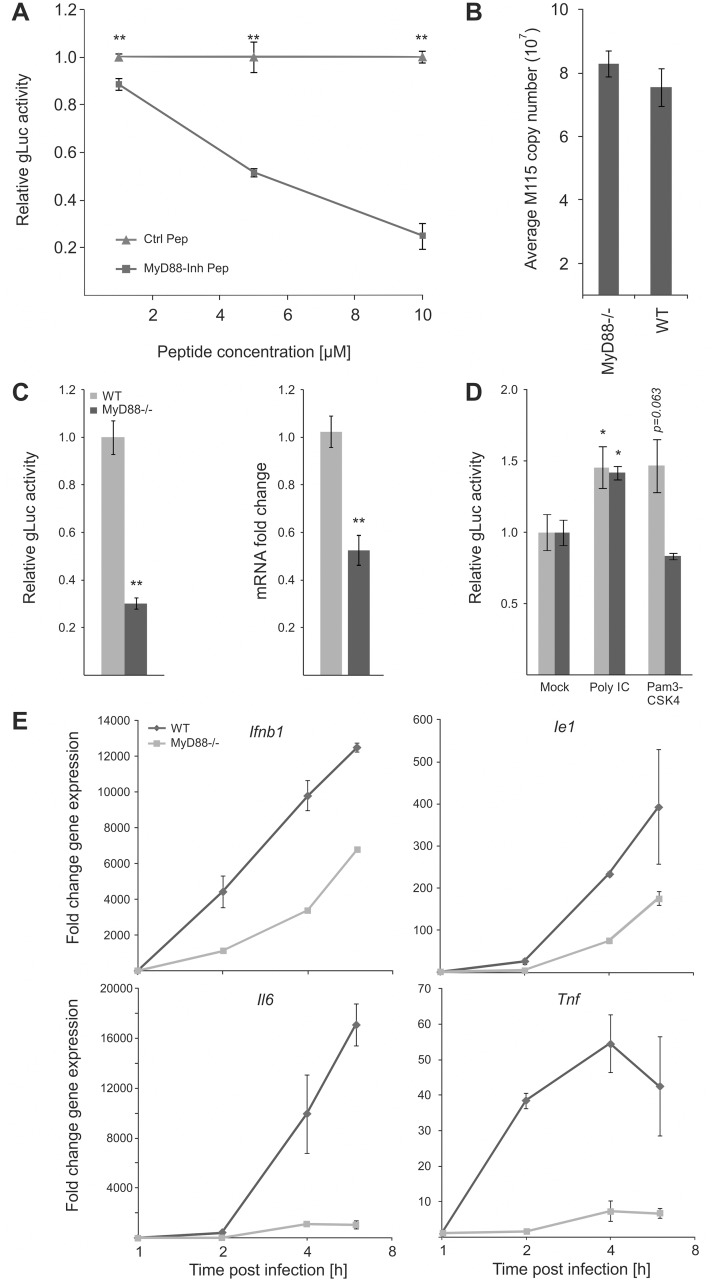
MyD88 governs MIEP activity. A) MyD88-specific inhibitor peptide reduces reporter gene expression compared to control peptide. MyD88 activity was inhibited in BMDMs by increasing concentration of a specific peptide inhibitor (MyD88-Inh Pep) prior to infection (MCMV-gLuc). Gluc activity was determined at 4 hpi and compared to the effects of a non-inhibiting control peptide (Ctrl Pep). Averages of normalised gLuc activity are shown (n = 6, with SEM). Asterisks indicate statistical differences in expression over vehicle (two sample Welch t-test; ** = p-value≤0.01). B) Loss of MyD88-signalling does not inhibit uptake of viral genomes. BMDMs from WT or *Myd88* ^-/-^ mice were infected with MCMV-gLuc and the number of intracellular viral genomes was determined at 6 hpi by absolute qPCR (n = 3). C) BMDMs from genetic knockout mice show lower MIEP activity. WT and *Myd88* ^*-/-*^ BMDMs were infected with MCMV-gLuc and reporter gene activity (left panel) and *Ie1* mRNA expression (right panel) was measured. Averages of normalised values f are shown with n = 15 (error bars show SEM) for gLuc activity and n = 7 for measurement of *Ie1* expression. Asterisks indicate statistical differences in expression over vehicle (Wilcoxon test; ** = p-value≤0.01). D) Induction of MIEP activity by TLR-agonists works through MYD88-dependent and—independent signalling. Stimulation with Poly IC (TLR3, MyD88-independent) is still capable to increase MIEP activity in *Myd88* ^-/-^ BMDMs while TLR2 stimulation (Pam3CSK4, MyD88-dependent) cannot increase MIEP activity in *Myd88* ^-/-^ cells. Averages of normalised gLuc activity are shown (n = 8, error bars show SEM) with asterisks indicating statistical differences over mock (Wilcoxon test; * = p-value≤0.05). E) Loss of Myd88 signalling reduces levels of viral Ie1 gene and host cytokine mRNA levels. Relative qPCR measurement of fold changes in expression levels for *Tnf*, *Il6*, *Ifnb1* and *Ie1* in WT and *Myd88* ^-/-^ BMDMs after infection (MCMV-gLuc). RNA was harvested at indicated time points from two infected cultures and measured in duplicates (n = 2). Expression levels were normalised to *Gapdh* and fold changes are relative to mock levels. *Il6* and *Ie1* did not show expression in mock samples so an arbitrary Ct value of 36 was chosen as reference point.

Our data show that all tested TLR ligands can boost viral enhancer activity and replication and consistent with this our functional network analysis identified MyD88 as a key hub. Therefore to further test and validate the results of the transient siRNA knockdowns in MEFs in our experimental BMDM system, we next treated BMDMs with a MyD88-inhibitor peptide at increasing concentrations before infection (Fig. 7A). These experiments showed that reporter gene activity was significantly inhibited in a dose dependent manner by the MyD88-inhibitor peptide but not by a scrambled control peptide. These results further corroborate the siRNA loss of function screens.

To unequivocally test the role of TLR-signalling and to establish the maximal impact of MyD88 on viral enhancer activity we next used BMDMs from genetic knockout animals (*Myd88* ^-/-^). Firstly, we characterised uptake of viral genomes for WT and *Myd88* ^-/-^ BMDMs by qPCR of intracellular viral genomes to eliminate the possibility that differential uptake causes differences in IE-gene expression (Fig. 7B) and found no reduction in the uptake of viral genomes in *Myd88* ^-/-^ cells. We next sought to confirm our results from the transient siRNA and peptide mediated inhibition in the genetic knockout system by infecting WT and *Myd88* ^-/-^ BMDMs with the gLuc reporter virus and measuring the reporter activity at 6 hpi. As shown in Fig. 7C (left panel) the loss of *Myd88* leads to a statistically significant (~70%) reduction of reporter activity. We also find a statistically significant reduction in endogenous *Ie1* gene expression levels as measured by qPCR under the same experimental conditions (Fig. 7C, right panel), excluding that this effect is an artefact of the reporter system. These experiments indicate that MyD88 is necessary for developing full levels of viral enhancer-activation upon infection.

Following this we further sought to determine how and to what extent the innate immune enhanced stimulation was specific and TLR-mediated. We compared the effects of a MyD88-dependent and MyD88-independent TLR ligand in WT and *Myd88* ^-/-^ BMDMs. As expected, stimulation of TLR3 by Poly IC significantly enhances viral IE-gene expression in both cell systems, while TLR2 activation by Pam3CSK4 increased viral IE-expression only in the WT but not in the MyD88-deficient cells (Fig. 7D).

### Loss of MyD88 co-ordinately affects expression levels of both, viral and host innate immune gene expression

Our results indicate that the host innate immune genes and the viral enhancer are governed by common factors and react to common stimuli. The observation that MyD88 is central and is a shared signalling factor regulating viral and host gene expression, suggests that the loss of MyD88 would impact on the host innate immune genes in a similar way as on the viral IE gene expression. To compare the impact of MyD88 on host and viral gene expression directly, we measured the expression kinetics of *Tnf*, *Il6* and *Ifnb1* with the viral *Ie1* gene in the context of the infection of *Myd88* ^-/-^ and WT BMDMs. Fig. 7E shows the comparison for each of the tested genes between the cell systems. The expression levels of viral and host genes are reduced in *Myd88* ^-/-^ cells compared to levels in WT cells. This data further supports the notion that induction of viral and host gene expression are governed by common host factors and that their expression is largely, but not exclusively, governed by a MyD88-dependent signalling pathway. It furthermore indicates that MyD88-dependent TLR signalling is necessary to achieve normal levels of IE-gene expression within the first 6 h post infection and shows that the boost in viral enhancer activity can be triggered by both, MyD88-dependent and -independent TLR signalling.

### Requirement of immune-activated transcription factor binding to the enhancer for viral gene expression and growth

**Fig 8:**
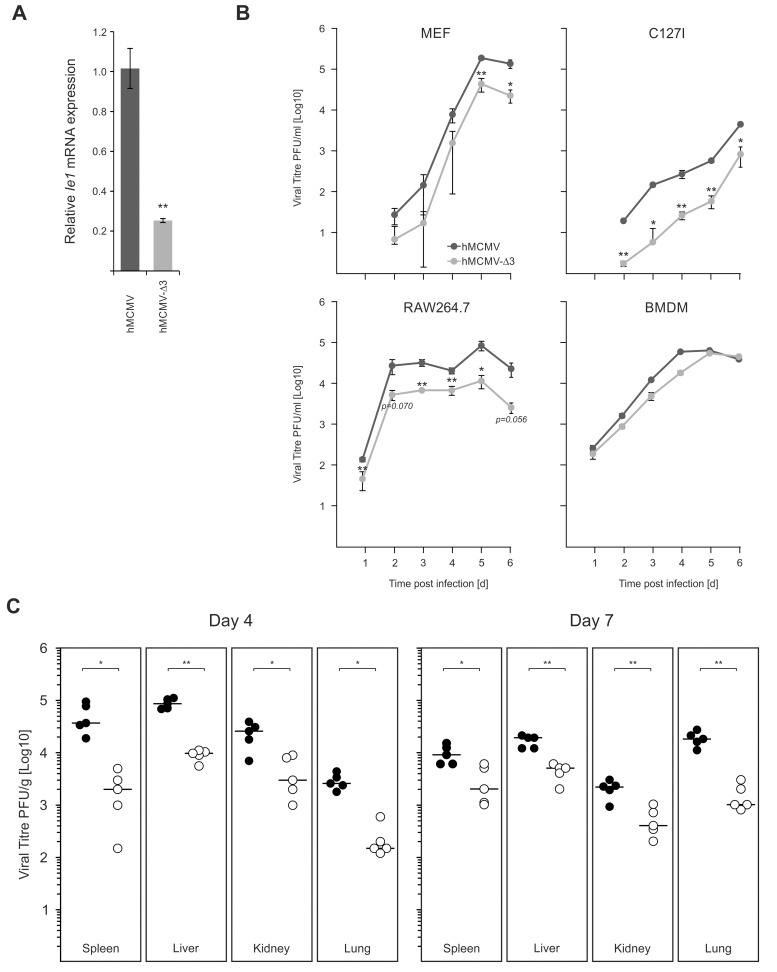
Loss of TLR-activated transcription factor binding motifs has impacts on viral replication and fitness. A) Comparison of viral IE-gene expression in hMCMV and hMCMV-Δ3 infected BMDMs (n = 4). Asterisks show statistical significant changes (two-sample Welch t-test; ** = p-value≤0.01). B) Comparison of in vitro replication of hMCMV and hMCMV-Δ3 virus in different cell types. Primary MEFs (MOI 0.025, n = 3), C127I (MOI = 0.025, n = 3), RAW264.7 (MOI 0.1, n = 3) or BMDMs (MOI = 2) were infected with either of the viruses and viral replication was measured by plaque assay at indicated time points on NIH3T3-Bam25 complementing cell line. Graphs show averages and error bars depict SEM. Asterisks show statistical significant changes (two-sample Welch t-test; * = p-value≤0.05, ** = p-value≤0.01). C) Comparison of viral fitness in neonate BALB/c mice. 3-day-old BALB/c females (n = 5) were i.p. inoculated with 5x104 PFU with either virus and sacrificed at indicated times post infection for plaque assay. Viral titres normalised per gram of tissue are shown (black circles = hMCMV; open circles = hMCMV-Δ3), with black bars showing median of groups. Asterisks indicate statistical significant differences between the two infected groups for each organ (two-sample Welch t-test; * = p-value≤0.05; ** = p-value≤0.01).

We next sought to characterise the role of key immune TLR-activated TFs for the virus and their contribution to viral replication. While we used Myd88^-/-^ macrophages to test for the effects on IE-gene expression, the impaired production of anti-viral cytokines, such as IFNβ and TNFα (see Fig. 7E) in this system would also influence viral replication and thus mask the effects of impaired TLR-signalling. Therefore, in an attempt to avoid this issue and to test directly the role of immune activated TFs, we analysed the IE-gene expression and *in vitro* and *in vivo* replication of the hMCMV-Δ3 mutant, which lacks the binding motifs for the major inflammatory TLR-activated transcription factors NFκB, AP1 and ATF that are known to bind the CMV enhancer. While we do not exclude the activation of these TFs by other signalling pathways, infection of NIH3T3 fibroblasts that are known to be insensitive to TLR agonists [81] with a M45 deletion mutant shows no activation of NFκB [36]. The *Ie1* expression in hMCMV-Δ3 mutant (Fig. 8A) reflected the effects we observed in the Myd88^-/-^ system (Fig. 7C, right panel), with a significant drop in *Ie1* expression compared to the hMCMV virus. To evaluate whether the enhancer point mutations affect viral growth we infected primary MEFs, the epithelial cell line C127I, RAW264.7 or primary BMDMs with the hMCMV and hMCMV-Δ3 viruses and measured production of viral progeny over 6 days (Fig. 8B). Notably, reduced levels of viral replication were detectable in multiple cells types. We next tested viral fitness of the two viruses *in vivo*. For these experiments neonatal BALB/c mice were intraperitoneally infected with either of the viruses and viral replication was monitored in the spleen, lungs, kidney and liver at day 4 and day 7 (Fig. 8C). In all tested organs and at both time points we found statistically significant reduction of infectious virus levels for the hMCMV-Δ3 mutant, indicating impaired viral fitness during an acute primary infection. Thus, while these experiments clearly show that the loss of the important immune regulated TFs NFκB, AP1 and ATF of the viral enhancer impacts on subsequent viral replication, *in vitro* and *in vivo*, we do not exclude contributions by other immune pathways, including possible activation by tegument proteins.

### The IRF TF-network: New candidates for TLR-induced host factors driving CMV-enhancer activity

The main target of the TLR-MyD88 signalling axis besides NFκB, is the family of IRF transcription factors. In our screening experiment we identified IRF5 as a member of the high stringency pro-viral network connected to the MyD88-dependent TLR pathway (Fig. 4 and 5A). A comparison of all IRFs in our siRNA screens showed that loss of the factors IRF4, IRF5 and IRF6 reduced gLuc activity and of these candidates, IRF5 knockdown had the most significant and strongest effect (Fig. 5B). This was an unexpected observation since IRFs are usually associated with driving the expression of type I interferons and other cellular defence factors such as NO after viral infection [7,82–85]. Furthermore, the HCMV tegument protein pp65 has been indicated to inhibit IRF3 activation after HCMV infection [24,25]. Firstly, to verify the efficiency of our siRNA knockdown approach, we measured relative expression levels for the IRFs in the context of infection and found that the siRNA treatment was sufficient to completely abrogate the up-regulation of IRFs 3, 5 and 7 and partially abrogate the induction of *Irf1* (S9 A Fig).

**Fig 9:**
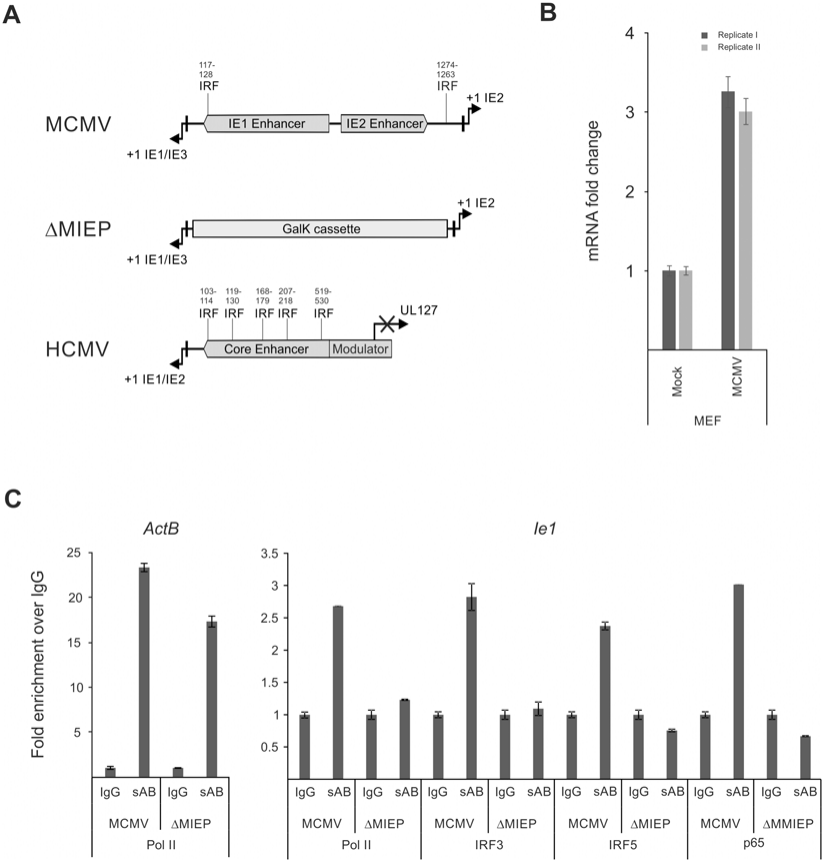
Family of IRF proteins represents new candidates for driving IE-gene expression. A) Sequence analysis of murine and human CMV enhancer regions shows potential IRF binding motifs with (~70% identity with canonical motif) at indicated bp positions (from +1 position of *Ie1/3* and *Ie1/2* respectively). Structure of the MIEP region in the MCMV-ΔMIEP recombinant is shown for comparison. B) *Irf5* is expressed in MEF cells. Relative qPCR data for *Irf5* expression, with mock-treated cells as calibrator. Cells were infected with MCMV for 6h before isolation of total RNA (n = 2) for qPCR. Samples were measured in technical replicates (n = 3, SEM). C) Representative ChIP experiment in infected RAW264.7 cells (MCMV, MOI 0.5, 24 hpi) with IgG as unspecific control and antibodies specific (sAB) for IRF3, IRF5, NFκB (p65) and RNA-polymerase II (Pol II), detecting the *ActB* and the viral MCMV enhancer (*Ie1*) by SYBRgreen qPCR measured in duplicates. Fold enrichment over IgG is shown and specificity was controlled by infection with the enhancer deletion mutant (ΔMIEP).

Next we asked whether these factors could interact with the CMV enhancer, therefore we analysed the human CMV and murine CMV enhancer sequences for potential IRF binding sites. We used the JASPAR binding motif database [86] to scan the viral enhancer sequences and were able to identify two potential IRF binding sites in the murine CMV enhancer and five potential binding sites in the human CMV enhancer (Fig. 9A). The level of sequence identity, with the IRF consensus binding motif (5’-AANNGAAA-3’), was relatively low with ~70% for both tested CMV enhancers. Work on *Irf5* has been focused so far on lymphoid immune cells such as B-cells and DCs [87–89] and therefore we asked whether *Irf5* is expressed in MEFs and in monocytes (RAW264.7) and if the *Irf5* expression in these cell systems is inducible by infection with MCMV. This experiment shows that we could detect *Irf5* expression in MEFs and that the expression could be further induced by MCMV infection. In the monocytic cell line RAW264.7 we could also detect higher levels of *Irf5* mRNA after infection, although the effect of the infection was lower than in MEFs (Fig. 9B, compare with S9 B Fig). However, we found that the RAW264.7 samples produced lower Ct values in the qPCR assay than the MEF samples, indicating that RAW264.7 cells have higher base levels of *Irf5* expression (average Ct 28.18 vs Ct 22.82, respectively). The expression data shown in Fig. 1B clearly indicates that *Irf5* expression is also inducible in BMDMs. These experiments demonstrate that *Irf5* is expressed in MEFs, BMDMs and RAW264.7 cells and that expression can be further induced in all our experimental systems by infection. It is therefore conceivable that IRFs might play a role in the direct activation of viral IE-gene expression.

To directly test whether the IRF sites in the MCMV enhancer interact with their cognate factors in the context of infection we performed a chromatin immunoprecipitation (ChIP) experiment firstly under low stringency conditions (S10 A Fig) using antibodies against IRFs 1, 3, 5 and 7 to pull down DNA-IRF complexes formed in infected RAW264.7 cells after 24 hpi. Since it is well established that NFκB has several binding sites in the CMV enhancer, we used an antibody specific for the p65 (RelA) subunit of NFκB as a positive control and also included a polymerase II subunit (Pol II) specific antibody to pull down transcriptionally active complexes. As a positive control of the pull-down we checked, if the Pol II antibody was able to enrich the host gene *ActB* over the unspecific IgG background control. When we used a primer pair spanning the TATA-box of the viral *Ie1* gene to detect enrichment of viral sequences, we could detect different levels of enrichment with the Pol II (~1.5x) and IRF3 (~2.1x), IRF5 (~1.2x) and p65 (~5.7x) antibodies over IgG. While similar results were obtained in an independent experiment, we cannot rule out the possibility that IRF1 or 7 may also interact with the enhancer region.

To further independently test the specificity of the interactions for IRF3 and 5 we next used a two-tier approach. First, we increased the stringency conditions in the experiment to further reduce unspecific background in the IgG sample. As shown in Fig. 9C the higher stringency conditions did not abolish but further enhanced the enrichment with the antibodies for Pol II (~2.6x), p65 (~3x) and IRF3 (~2.8x) and IRF5 (~2.4x) over IgG for the viral sample. The enrichment of IRF3 and 5 was consistently observed in other independent experiments (S10 B Fig). As a functional positive control for the antibodies, we also measured enrichment of the established inflammatory and IRF-controlled genes *Cxcl10* and *Ifnb1* and we could detect enrichment with Pol II (~9x), IRF3 (~2x) and p65 (~3x) antibodies for *Cxcl10* but only minor enrichment for Pol II (~2x) and IRF3/p65 (~1.2x) for *Ifnb1*. Second, as an additional control for the specificity of the pull-downs, we used a mutant virus lacking the enhancer sequence (MCMV-ΔMIEP) to assess if a loss of the binding sites in the MIEP region of the viral genome also leads to a loss of the enrichment in the ChIP experiment (Fig. 9C). The level of enrichment for the *ActB* control gene in the MCMV-ΔMIEP sample was lower than in the wild-type MCMV infection but overall similar. The pull-down with the Pol II control antibody produced a smaller enrichment of the viral MIEP sequence with the MCMV-ΔMIEP sample than in the MCMV infected sample, which corresponds well with the overall drop in gene expression in a MCMV-ΔMIEP recombinant [42]. When we compared the enrichment of viral MIEP sequence for the IRF5 or p65-binding antibodies we could not detect any enrichment (level less than IgG control), while we detected a minimal increase in the IRF3 sample. These data show that the observed enrichment was reproducible and specific, since loss of TF binding sites abolishes the interaction of the host factors with the viral sequence and therefore the enrichment in the ChIP experiment. Further studies will be required to more precisely define the role of IRFs in the regulation of the enhancer.

Taken together these data highlight the IRF protein family as representing a new group of host factors that target the MIEP within the first hours of infection. These factors are directly driven by TLR signalling and are involved in driving the expression of important anti-viral factors. Hence these results provide a direct molecular link outside NFκB for the co-option of the TLR signalling pathway and TF network by CMV and suggest a new strategy for CMV to stay ahead of the anti-viral response.

## Discussion

The initial interactions between host and pathogen trigger PRR-signalling that lead to the production of inflammatory antiviral cytokines and innate immune effector molecules, such as type I interferons [2,90]. Activation of these inflammatory signalling pathways is therefore generally considered to be detrimental for the pathogen. To ensure successful initiation of viral gene expression and replication in the case of DNA nuclear viruses two main strategies are employed: 1) inhibition of the host signalling molecules and TFs to prevent initialisation of an anti-viral state or 2) co-opting the activated signalling molecules to ensure a rapid initiation of viral gene expression to stay ahead of the production of antiviral factors [91]. Of the central TFs activated by canonical TLR signalling pathways [47, 90], NFκB, AP1 and CREB/ATF are known to bind and regulate a wide range of viral enhancer/promoter sequences [47]. NFκB is a central inflammatory transcription factor that controls expression of innate immune genes [2,3,83,92] and is hijacked by several viruses (as reviewed in [93]). It has been shown that TLR8-mediated activation of NFκB is necessary for replication of HIV1 [94] and the human CMV and murine CMV [50,51,95–97]. However, when we assessed the contribution of these factors to the observed effects of TLR stimulation we found that the pro-viral effects were markedly reduced but not completely abolished. Therefore other TLR-activated TFs had to be involved in the observed phenotype. We used a siRNA screening approach to systematically investigate host factors. This loss-of-function screen indicated that the overlap in shared TFs is considerably more extensive than so far recognised, identifying novel potential interactions with host factors including several IRFs. In addition, knockdown of a SOX gene reduced viral gene expression. We initially included this gene family as a negative control since SOX genes are mainly associated with developmental genes [98,99]. However, a subsequent bioinformatics analysis [86] of the MCMV enhancer showed several potential binding sites for SOX proteins. Also notable is the presence of YY1 as a hit in this screen since YY1 so far is associated with inhibition of human CMV [100]. Furthermore, knockdown of STATs did not show an anti-viral effect in our system [101]. We also found retinoic acid receptor RAR-RXR to be part of the integrated TF network. While retinoids are known to directly regulate human and murine CMV enhancer activity [74,77], our results suggest both direct binding of RARE motifs on the viral genome and possible indirect mechanisms including changes in TLR expression through retinoic acid [78,79] and possible alteration of NFκB binding kinetics to the viral enhancer [102].

We identified IRFs 4, 5 and 6 as potential novel pro-viral host factors. IRF4 is a lymphocyte specific factor [103] and the IRF6 gene is largely uncharacterised except for an association with van-der-Woude-syndrome [104]. We therefore focused on IRF5 as it also showed the strongest effects in our functional experiments. Since the first study characterising IRF5 [85] it has been associated with systemic lupus erythematosus [105] and plays an important role in controlling type I IFN expression [58,85,89]. It is a central component of the TLR7 signalling pathway [106] but also part of TLR3, 4 and 9 signalling and is therefore target of MyD88 and TRIF/TBK1-dependent TLR signalling [58]. Notably, in some cell systems IRF5 is directly activated by TRAF6, therefore forming a signalling bypass around the highly controlled and modulated NFκB pathway [87,107,108]. As a result of its role in controlling the IFN anti-viral response, IRF5 is blocked by Newcastle Disease Virus [109] and Epstein-Barr Virus [110]. Considering that we observed the knock-down of *Irf5* to have a strong effect in the enhancer screen, we tested if the CMV enhancer is capable of directly interacting with the activated IRF proteins by ChIP. We observed interactions of IRFs 3 and 5 with the viral enhancers that have not been previously documented, however other IRFs have been reported to interact with enhancers of HPV-16 [111], HBV [112] and HIV-1 [113]. Notably, it has recently been shown that IRF3, IRF5 and IRF7 co-ordinately regulate type I interferon production downstream of RIG-I [114], of which retinoic-acid induced gene-1 (RIG-I) and IRF3 and IRF5 are statistical significant hits in our enhancer activation screen, implying that other PRR signalling pathways might contribute to the initial activation of the viral enhancer. Further studies will be required to more fully characterise the IRF family interactions with the CMV enhancer. Nonetheless, the engagement of IRFs appears to be a general feature for a wide range of viral enhancers. However, in contrast to these other studies we delineate the time restricted dependency on TLR signalling that is exploited to the advantage of the virus.

Concordant with inflammatory signalling promoting infection, we observed comparable expression kinetics for the immune genes *Il6*, *Ifnb1* and *Tnf* in comparison with the viral gene *Ie1* with microarray experiments extending this finding to a larger class of known innate immune genes, although not all of the identified genes belong to the immediate early immune response class. These results indicate that the similarity in the expression kinetics of the host immediate response genes and the viral IE-genes is likely based on the same inflammatory mechanisms. This notion is further supported by the experiments in the genetic knockout system for the signalling adapter *Myd88* that equally affected cellular and viral gene expression.

**Fig 10:**
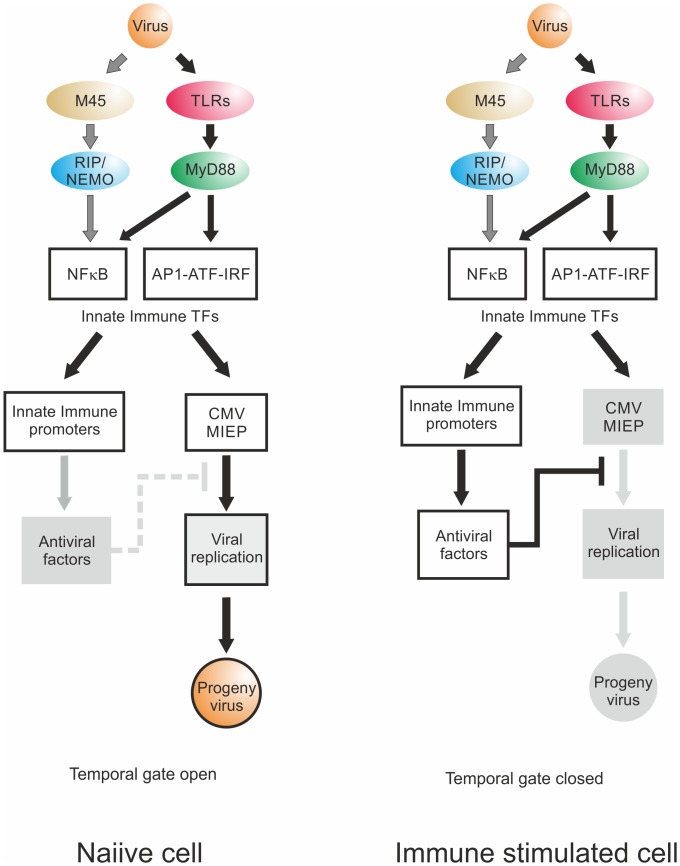
Temporal gate model. Virion delivered M45 and PRR signalling activate NFκB and other TFs downstream of TLRs, leading to transcription of innate immune effectors and viral IE-gene expression. Temporal gate open: In a *naiive* cell, viral infection triggers innate immune effector expression but at the same time drives viral gene expression. By the time host innate immune effectors are produced and can act on the viral IE-gene expression and replication, the virus already has progressed through this checkpoint of replication, produced viral inhibitors of innate immune signalling such as UL26, M27 or M45 and, using this lag-phase, escapes host control. Temporal gate closed: In a non-*naiive*, immune stimulated cell, the anti-viral effectors are already active at the point of infection and directly inhibit viral IE-gene expression. The viral gene expression cannot effectively progress beyond the IE-phase and the viral infection is controlled; the temporal gate is therefore closed.

While long term stimulation of TLR signalling is associated with inhibition of viral gene expression and replication, we found that short-term stimulation of the TLR signalling pathway, just prior to infection, can actually boost viral IE-gene expression. This indicates the existence of a temporal gate in which CMV can exploit TLR signalling before it initiates anti-viral effects (illustrated in Fig. 10). We have previously characterised the involvement of Type I interferon inhibition of the CMV enhancer in our macrophage system with IFNβ protein levels peaking by 6 hpi in BMDMs [64]. In the present study we define a temporal gate for TLRs 4 and 9, as being open for murine CMV to co-opt TLR-signalling with a positive effect within ≤ 6 hpi and a negative effect for >6 hpi, which correlates well with known timing of TLR signalling and the induction of TLR controlled gene expression in various systems [115–117] and the induction of ISGs by HCMV [118]. This indicates that a *naiive* macrophage needs optimally 6 h to establish a full anti-viral state after activation of TLR signalling.

We find that the TLR ligands had differential effects. In the BMDM system, only ligands for TLR2, TLR3 and TLR4 triggered an increase of IE-gene expression. In support of this finding it has been described before for human CMV that glycoprotein B on the viral particle surface can bind to TLR2 and that the interaction with this receptor is sufficient to activate NFκB and to induce production of pro-inflammatory cytokines [12,13,92,119]. This interaction is conserved within Herpesviruses, since the gB protein of HSV1 can also interact with TLR2 and activates NFκB, via a MyD88/TRAF6 dependent signalling pathway [14,120]. This indicates that the gB-TLR2 interaction might be the result of evolutionary selective pressure, since this interaction has the most beneficial impact on CMV replication with the longest determined temporal gate, the weakest inhibition and the largest boost in progeny virus production.

Besides triggering of host PRR-signalling pathways by CMV leading to the activation of NFκB it has been recently demonstrated that the viral protein M45 mediates NFκB activation immediately after infection in NIH3T3 fibroblasts [36]. This finding is in contrast to the NFκB inhibitory function M45 has during the early and late phase of the infection at >5 hpi [34–36]. Notably, in macrophages and endothelial cells M45 is not essential for NFκB activation and IE1 protein levels. TLR agonist mediated stimulation of *Ie1* expression was not impaired in cells infected with M45 negative virions, showing that both ways of activating NFκB operate independently.

Taken together, our data show for the first time a direct dependency of the CMV enhancers on MyD88-dependent TLR-signalling to ensure the maximum expression of the essential IE-genes. We identified IRF3 and IRF5 as novel pro-viral factors within a specific temporal gate which shows that CMV is not limited to exploiting NFκB and that many innate immune TFs could contribute to viral IE-gene expression. This strategy allows the CMV expression program to stay ahead of the anti-viral state in the infected cell. We therefore propose a model (schematically shown in Fig. 10) incorporating a temporal component for infection of *naiive* cells, in which a CMV virion exploits the TLR response through the downstream transcription factors NFκB, AP1, CREB/ATF and IRFs, additionally to and independent from the activation of NFκB by virion delivered M45. These activated TFs then facilitate rapid viral IE-gene expression initiating the viral replication cycle before the cell can express any anti-viral effector molecules. By the time the cellular anti-viral factors (e.g. IFNβ) start to inhibit the viral IE-gene expression, the viral replication already progressed through this checkpoint, expresses viral modulators of signalling and the virus escapes host control. This is likely to be a common mechanism for a wide range of DNA viruses including retroviral enhancers such as those of HIV [47]. Since the exact composition of the activated TFs downstream of the TLR signalling pathway differs dependent on cell type, functional mimicry of a combination of host innate immune enhancers by viral enhancers in general would provide flexibility to harness activation of different innate immune related TFs in the context of different cell systems.

## Materials and Methods

### Cell preparation and culture

Primary mouse embryonic fibroblasts (MEFs) were prepared from C57/BL6 embryos at gestational age day 10–14 as described in [121]. Immortalized murine embryonic fibroblasts (MEF) used in the western blot experiments were provided by Edward Mocarski (Emory University, Atlanta, GA). MEFs were cultivated in MEM (Lonza) (+ 2 mM L-glutamine, 100 U Penicillin/Streptomycin (Pen/Strep) and 10% Fetal calf serum (FCS, Lonza) for 3 passages before being used for any experimental procedure. Bone marrow derived macrophages (BMDMs) were prepared from bone marrow of C57/BL6 mice and differentiated in DMEM F-12 GlutaMAX (Lonza) (+Pen/Strep, 10% L929 conditioned medium (contains Csf1) and 10% FCS) for 7 days. Efficiency of differentiation medium was analysed by FACS, testing for surface markers C11b and F480. MyD88^-/-^ BMDMs were prepared from MyD88^-/-^ homozygous knock-out animals [122] on C57/BL6 background, provided by the MacDonald group in the Institute of Immunology & Infection Research, University of Edinburgh, UK. C57BL/6 (BL6) mice were purchased from Charles River Laboratories (Kent, United Kingdom) and maintained under specific pathogen-free conditions at the University of Edinburgh. BALB/cOlaHsd mice were obtained from Harlan (Netherlands) and housed in the vivarium (University of Barcelona). All procedures were carried out under project and personal licences approved by the Secretary of State for the Home Office, under the United Kingdom’s 1986 Animals (Scientific procedures) Act and the Local Ethical Review Committee at Edinburgh University and by the Ethics Committee (protocol number CEEA 308/12) of the University of Barcelona (Spain) and were conducted in compliance with institutional guidelines as well as with national (Generalitat de Catalunya decree 214/1997, DOGC 2450) and international (Guide for the Care and Use of Laboratory Animals, National Institutes of Health, 85–23, 1985) laws and policies. All cultures are routinely tested for mycoplasma and endotoxin levels.

IC-21 macrophages ATCC TIB-186), RAW264.7 (ATCC TIB-71), RAW264.7 for the western blot experiments (ATCC CRL-2278), C127I (ATCC CRL-1616) and NIH3T3 (ATCC CRL-1658) cells were obtained from the American Type Culture Collection (Manassas, VA) and were cultivated in DMEM (Lonza) supplemented with 100 U Pen/strep and 2 mM L-glutamine and either 10% FCS (RAW264.7 and C127I cells) or 10% CS (NIH 3T3 cells). All cells were cultivated under standard tissue culture conditions. The Institute of Animal Breeding and Genetics, Department provided STAT1-/- fibroblasts for Biomedical Sciences, University of Veterinary Medicine Vienna, Austria.

### Viral strains and infection procedures

Viral infections, growth procedures and plaque assays were carried out as described in [121,123]. In short, to infect cells volume of culture medium was reduced depending on the culture size and viral particles were added, pre-diluted in growth medium, to an MOI of 0.1 if not stated differently, followed by incubation for 1 h at 37ºC for adsorption of viral particles. Cells were subsequently washed briefly 1x in medium after adsorption. To produce viral stocks NIH3T3 cells were infected at low MOI with a viral seed stock and subsequently infectious viral particles were harvested at day 4 for further concentration and titred by plaque assay.

Virus reconstituted from the bacterial artificial chromosome (BAC) pSM3fr was used as wild type MCMV [124]. The fluorescent MCMV-GFP reporter virus used for viral replication assay [125] has been described before. In this study we used a previously described viral mutant [36] lacking the entire M45 open reading frame (ORF) that was generated by *en passant* mutagenesis, here named MCMV-ΔM45. The viral gLuc-mutant MCMV-gLuc was constructed by homologous recombination [126] with a bacterial artificial chromosome (BAC) carrying the viral genome [124,127]. The mutation strategy is summarised in S1 Fig. In short, the reporter cassette containing the bicistronic ORF for the Gaussia luciferase (*gLuc*) and GFP genes and the kanamycin resistance gene was constructed by splice-PCR [128], including homologous sequences at the 5’ and 3’ ends to enable the replacement of the non-essential *Ie2* gene in the BAC by homologous recombination. The GFP gene was amplified using primers GFP-HOMO-FOR1: 5’-GACGGACCGCGGCTCGATACGACCCTATCTACGTTAACGA-**ATGGTGAGCAAGGGCGAGGA**-3’ and GFP-P2A-REV3: 5’-CGGCCTGCTTCAGGAGGCTGAAGTTCGTGGCTCCCGAGCC-**CTTGTACAGCTCGTCCATGC**-3’ with viral DNA from strain MCMV-GFP as a template (bold stretches indicate primer sequence complementary to template sequence). The *gLuc* reporter gene and the kanamycin reporter gene were amplified using the primers P2A-FOR: 5’-CTCGGGAGCCACGAAC-3’ and GLUC-HOMO-REV2: 5’-GAATAAAACCTCTTTATTTATTGATTAAAAACCATGACAT-**CGCCAAGCTAAGCTTGGATC**-3’ with the plasmid mCMV-mcherry-gLuc-kanamycin-puc19 [64] as a template. The two products of these reactions were then spliced together by using them as primers/template in the first 5 cycles in a splicing PCR [128]. After the first 5 cycles primers GFP-HOMO-FOR1 and GLUC-HOMO-REV2 were then added to the reaction for further 30 cycles to amplify the full-length cassette. The final product contained therefore 40bp stretches of sequence homologies to the viral BAC, allowing for targeted replacement of the viral *Ie2* gene by homologous recombination.

For construction of the enhancerless MCMV-ΔMIEP reporter virus, the viral enhancer sequence was replaced from transcription start of the *Ie1* gene to the transcription start point of the *Ie2* gene with the galactose kinase (GalK) ORF, allowing for selection by carbon source [129]. The GalK ORF was amplified using primers GALK-BAC-FOR: 5’-CGCCTCTTATACCCACGTAGAACGCAGCTCAGCCAATA-**CCTGTTGACAATTAATCATCGG**-3’ and GALK-BAC-REV: 5’-GCTTTTATATGGTTAACTCCGCCCGTTTTATGACTAGAAC-**TCAGCACTGTCCTGCTCCTT**-3’ with the plasmid pGalK as a template molecule. Restriction digests with BamHI and EcoRI and PCR assessed successful recombination and integrity of the BAC and viral genome. The mutant MCMV-gLuc was reconstituted by transfection of NIH3T3 cells with isolated BAC DNA and infectious particles were harvested and further propagated. For the MCMV-ΔMIEP virus the complementary cell line NIH3T3-Bam25 was used to allow for reconstitution and propagation of this growth deficient viral mutant as described before [54,125].

For the generation of hMCMV-ΔRARE and hMCMV-Δ3 mutants, the hMCMV-ES.RARE BAC and hMCMV-ESNFkB/Ap1/ATF BAC, respectively, were constructed by the two-step mutagenesis procedure as described in [50] using the MCMV pSM3fr BAC [124] and the shuttle plasmid pST76-AsacB.MIEP.RARE for the construction the hMCMV-ES.RARE BAC, and the MCMV enhancerless C3XdE [39] and the shuttle plasmid pST-ES.NFkB/Ap1/ATF for the construction of hMCMV-ESNFkB/Ap1/ATF BAC. Vector pST76-AsacB.MIEP.RARE carries the four RAR/RXR binding sites [76] present in the 613-bp HCMV enhancer region (from nt -52 to nt -667) disrupted by site directed mutagenesis. pST-ES.NFkB/Ap1/ATF contains the two AP-1 [69], the four NF-kB [50], and the five ATF/CREB binding sites within the 613-bp HCMV enhancer abolished by site directed mutagenesis (S5 Table). ATF/CREB binding sites disrupted in pST-ES.NFkB/Ap1/ATF were located at enhancer positions from -464 to -457, from -410 to -403, from -328 to -321, from -142 to -135, and from -66 to -59. The resulting BACs were transfected in NIH3T3 cells. Progeny virus obtained from the transfections were amplified, subjected to three rounds of plaque purification, and used for the preparation of viral stocks. The integrity of the viruses generated was confirmed by restriction enzyme analysis, and enhancer regions were sequenced.

### Viral growth assays in neonate BALB/c mice

Three-day-old female BALB/cOlaHsd mice were inoculated intraperitoneally (i.p.) with 5x 10^4^ PFU of tissue culture-propagated hMCMV or hMCMV-Δ3. At designated times after infection, mice were sacrificed, and specific organs were removed and harvested as a 10% (weight/volume) tissue homogenate. Tissue homogenates were sonicated and centrifuged, and viral titers from the supernatants were determined by standard plaque assays, including centrifugal enhancement of infectivity on MEFs.

### TLR ligand pre-treatment

For stimulation of TLR-signalling, cells were incubated with 5 ng/ml LPS from *E*.*coli* 0111:B4 (Sigmal-Aldrich, L2630), 10 μg/ml Poly IC (Invivogen, Tlrl-pic), 100 nM Pam3CSK4 (Imgenex, Img2201), 100 nM R848 (Alexis, ALX-420-038-M005) or 100 nM ODN1668 (Invivogen, tlrl-1668) in cell culture medium for indicated pre-treatment times. After pre-treatment, cells were carefully washed with medium 1x and subsequently used for infections or other experiments.

### 9-*cis* pre-treatment

For pre-treatment with 9*-cis* retinoic acid (9*-cis* RA, Sigma-Aldrich UK) cells were incubated with 1 μM 9*-cis* RA for 24h before being used in subsequent experiments. Stock solution was 1 mM in DMSO and 9*-cis* RA working solution was prepared with medium immediately before use. 9*-cis* RA stock solutions was handled under reduced light conditions and stored at -80°C under Argon gas. A DMSO solution as a vehicle control was used with the corresponding dilution.

### BAC-DNA transfection

MEFs were seeded in 48-well plates to reach ~90% confluency on the day of transfection. For transfection (per well) 250ng of BAC DNA and 0.75ul JET-PEI (PolyplusTransfection, Illkirch, France) were used as described in supplier’s instructions. DNA-transfection complexes were dripped onto cells and system was left for recovery o/n (16h) before experiments.

### Viral entry assay

To measure entry of viral particles, BMDMs were seeded (1x10^5^ cells/well) in 24 wells. The next day, cells were infected with MCMV (MOI = 0.5) for 1 hr. After adsorption, cells were washed 3x with medium, trypsinised for 5 min and scraped off the culture plates for lysis and DNA isolation. Viral genomes were measured by absolute qPCR of viral ORF M115. To establish effectiveness of Trypsin treatment, cells were infected as described above and subsequently either washed A) 4 times with PBS, or B) 1 time with PBS, tyrpsinised for 1 min, then washed twice with PBS, or C) 1 time with PBS, washed with citric acid buffer (40mM citric acid, 10mM KCL, 135mM NaCl) for 1 min, then washed twice with PBS. DNA was extracted using the Qiagen Qiaamp mini kit (51304) protocol and the copy number of the virus was determined by measuring M115.

### Microarray data analysis

For Affymetrix Mouse Gene 1.0 ST Microarray analysis, data from study [130] was processed using the PARTEK software Package. In short, expression data was RMA normalised (quantile normalisation) and subsequently normalised per gene to average signal intensity over the time course (mean = 0.0, SD = 1.0). We then sorted the expression profiles for all genes by their similarity to a generated profile displayed by the genes in Fig. 1 A. The profile used showed no expression in the mock, rapid induction of expression within 2 h, a peak expression between 4 and 6 h and a drop in expression levels at 8 h. Gene expression profiles were sorted by similarity to this profile using a Bayesian statistical clustering function, identifying a large number of immune related genes in the cluster with the highest similarity including the selected 37 known innate immune genes used in this study.

### Small molecule inhibitors

#### Gene knockdown by siRNAs

For transient siRNA mediated gene knock-down of host cells primary MEFs were reverse transfected with Dharmacon SMARTpools (Life Technologies) as described in manufacturers manual. In short, siRNAs were plated (final concentration of 25 nM or 2.5 nM) in siRNA buffer into 96-well tissue culture plates (Black plates clear bottom, Costar), and were then mixed with Dharmafect 1 transfection reagent (0.4% final concentration) diluted in OPTIMEM (Invitrogen). After incubation for 20 min at RT to allow formation of transfection complexes a cell suspension (2x10^4^ cells/well) in antibiotic free medium was added. Cells were then incubated for 48 h after transfection to allow for protein depletion before being infected with either the GLuc-MCMV or the GFP-MCMV reporter viruses to assess IE-gene expression or viral replication respectively. Short interfering RNA duplexes designed to target the gLuc reporter (TCAAAGAAATGGAAGCCAA), the viral DNA polymerase M54 (AGAAAGACGACCTGAGCTA) or the viral major capsid protein M86 (CGACGGAGCTGCTGCCTAA) were designed using the Dharmacon siRNA design centre software and used as positive controls for gene knock-down and viral inhibition.

#### MyD88-inhibitory peptide

For transient inhibition of MyD88 protein function a 26aa (RQIKIWFQNRRMKWKK-RDVLPGTCVNS-NH2) inhibitory peptide (Pepinh-MYD) was used (Invivogen, tlrl-piMYD). The first domain of the peptide is a sequence that allows it to translocate through the cell membrane and the second domain (underlined in above sequence) binds and blocks the function of the TIR domain. As a control a peptide with a translocation sequence followed by a non-interacting domain was used (RQIKIWFQNRRMKWKK-SLHGRGDPMEAFII-NH2). For inhibition of MyD88 activity, BMDMs were pre-treated with increasing amounts (1–50 μM) of the inhibitor or the control peptide for 6 h before using them for subsequent experiments.

### Gaussia luciferase enzyme assay

The gaussia luciferase enzyme reporter assays were performed as described elsewhere [64]. In short, cells were infected with the reporter virus at MOI 0.1 if not stated differently (adsorption for 1h at 37°C) and then washed 2x with medium. Infected cells were then incubated for indicated time windows and complete culture supernatant was then repeatedly harvested. After each sampling time point cells were re-fed with fresh medium and further incubated as indicated in the respective experiments. To measure enzyme activity, 50μl of sample were mixed with 50μl fresh substrate working solution (20nM native coelenterazine in PBS + 5M NaCl) and light emission was measured using a POLARstar plate reader (BMG Labtech, Germany).

### Immunoblotting

Viral titers were determined using the median tissue culture infective dose (TCID_50_) method [131]. Infections were carried out with centrifugal enhancement (1,000 × g, 30 min). For infection kinetics, cells were grown in 12-well dishes, infected at an MOI of 10 TCID_50_/cell, lysed in SDS-PAGE sample buffer, and subjected to SDS-PAGE and immunoblotting.

#### Antibodies

Monoclonal antibodies against M45 (M45.01 mAb, provided by Stipan Jonjic, University of Rijeka), IE1 (Chroma101; provided by Stipan Jonjic) and β-Actin (AC-74; Sigma) and polyclonal antibodies against IκBα (C-21; Santa Cruz) and M45 [132] (provided by David Lembo, University of Turin, Turin, Italy) were used.

### Chromatin Immunoprecipitation (ChIP)

To analyse DNA-protein interactions RAW264.7 cells or primary MEFs, cells were infected at MOI 0.5 for 24 h before cells were used for chromatin immune-precipitation as described before [133,134]. In short, cells were fixed in 1% formaldehyde for 10 min (stopped with 0.125 M Glycine) and directly used for sonication of chromatin. Sonicated chromatin was aliquoted and stored at -80°C until used for pull-downs. For the immune-precipitation a mix of magnetic beads coated with protein A and G (Dynalbeads, Invitrogen) were coupled to 5μg primary antibody (per pull-down) and were used with 100 μg of chromatin o/n at 4ºC to precipitate DNA-protein complexes. For low stringency runs, the precipitated complexes were washed 4x for 3 min at 4ºC on a rotator with LiCl buffer (250mM) and 2x with Tris-EDTA (TE) to remove unbound chromatin. For high stringency washes, protocol was adjusted, considering procedures described in [135]. Complexes were pre-cleared by incubating them with uncoupled protein A/G Dynalbeads on a rotor for 1 h at 4ºC before performing the immune-precipitation. For high stringency washes complexes were incubated 4x for 10 min at 4ºC with LiCl buffer (500mM) and 2x with TE before reversing crosslinking and elution from beads. Used antibodies for ChIP experiments: IgG (Sant-Cruz, rabbit IgG sc-2027), Pol II (Abcam, 8WG RNAPII: Covance RNA Polymerase II 8WG16 Monoclonal- MMS-126R), NFκB (p65, AbCam, Ab7970), IRF1 (Bethyl, Rabbit A303-376A), IRF3 (Bethyl, Rabbit A303-383A, IRF5 (Bethyl, Rabbit A303-386A), IRF7 (Abcam, ab62505).

Eluted DNA was cleaned up using a Qiaquick PCR purification kit (Qiagen, 28104) and genes of interest were detected by qPCR using a Quanta PerfeCTa Sybrgreen Mix (95073) and primers detecting either ActB as a control gene or the CMV *Ie1* transcription start site (Enh1_L 5’-CGCCTCTTATACCCACGTAGA-3’; Enh1_R 5’-CACGTCAATGGGAAGTGAAA-3’) with a relative standard curve produced from input DNA. To detect DNA of the ΔMIEP mutant the primers Enh1_L and dEnh_gLuc_R (5’-CTGCCTCCTGGGTTTAGTT-3’) were used. Quantities were measured relative to input DNA and enrichment of target sequences was calculated relative to the signal in the unspecific IgG-antibody pull-downs.

### Quantitative real-time PCR

To measure relative gene expression from total cellular RNA we used FAM-labelled AB gene assays for host genes (Tnfa: Mm00443260_g1, *IL6*: Mm99999064_m1, Ifnb1: Mm00439546_s1) in duplex with a VIC-labelled GAPDH assay (Mm99999915_g1) for normalisation and the Quanta TOUGH-mix (Quanta, 95123) ready-to-use qPCR master mix. For detection of the viral *Ie1* gene expression we used a custom probe and primers described previously [136]. Expression levels were measured with a Stratagene Mx3000P qPCR machine (Agilent) and fold changes were determined by the ΔΔCt method using the MxPro software package.

For absolute quantification of viral genomes in infected cells or in culture supernatant, DNA was isolated using the Qiaamp mini kit (Qiagen, 51304) and subsequently used in absolute quantification by quantitative PCR. To quantify viral genomes we used a custom gene assay from AB (1166810B8) specifically designed to detect the viral M115 gene. A standard curve of a linearized plasmid carrying the M115 gene was used for absolute quantification as described previously [54,137].

### Statistical meta-analysis

Meta-analysis of siRNA screens was performed to combine the results of multiple independent screens under similar biological conditions. Meta-analysis was applied separately for siRNA screens conducted with a GFP readout and for siRNA screens with a gLuc readout. The GFP data set comprises a total of 24 studies, where a study is defined as an independently performed siRNA screen on a 96-well plate containing multiple knockdown and control replicates. The gLuc data set comprises 10 studies. The number of within-study replicate knockdowns and controls (i.e. wells) differs from study to study and is detailed in S4 Table. For each study (screen on plate), the average fold change between each siRNA knock-down and the infected control was calculated, with average computed on the Log_2_ scale and the fold change computed as difference = mean (siRNA)-mean (infected control). The robustness of the fold change estimates will depend on the presence and number of replicate measurements as shown in S4 Table. The fold change estimates are considered as the per study effect sizes. For each siRNA, these effect sizes are then combined across studies by an un-weighted median, and the standard error of this median estimated through b = 1000 bootstrap samples of the data for a given siRNA. For each siRNA, the significance of this median fold change is then tested by a two-sided location test (Wilcoxon Signed Rank Test) against the underlying Null hypothesis that the distribution of the fold change values is symmetrical around zero. The statistical power for each analysis will depend on the number of studies a given siRNA has been measured in (not all siRNAs are present in all screens). For the GFP set the overall n for siRNAs is 4, 6, 8, 10, 12, 18 or 24. For the gLuc overall n is 3, 4, 7 or 10. For each siRNA, the corresponding n is indicated in Fig. 5 by vertical bars along the x-axis. With results intended as an interest filter for further validation, p-values were not adjusted for multiple testing on these 184 siRNAs simultaneously. Differences in replicate number per study and number of studies available per siRNA suggest limited reliability of the meta-analysis outcomes and are therefore used as an interest filter in conjunction with corroborating evidence from subsequent validation experiments.

### Statistical analysis

Inference testing for each experiment is applied in all cases where the number of independent biological replicates is 3 or higher. This consists of assessing underlying data distributions (Shapiro-Wilks test [138]), removing any objectively extreme outlier observations (Grubbs’ test [139]) and subsequent retesting for the remaining data distribution. In experiments where no observation groups deviate from a normal distribution, 2-sample (or 1-sample where the comparison is to a constant value) Welch t tests were used to compare experiment groups. In experiments where one or more observation groups significantly deviate from a normal distribution, the non-parametric equivalent (Wilcoxon Rank Sum Test) was used to compare experiment groups. Note that all experiment figures show arithmetic means and standard errors of means, irrespective of the statistical test being parametric or non-parametric. The type of test performed and associated groups sizes are stated in each figure legend. All statistical analyses were performed with R [140].

### STRING pathway analysis

For detection of networks in the screening data we used the STRING [71] online tool (v9.05, http://string-db.org). Hits were sorted by % of maximum knockdown effect (gLuc control siRNA = 100%) and cut-off levels of 25%, 50% and 75% of maximum effect were applied. List of gene names were then imported into STRING and identity of the factors manually checked. String association networks were built using standard settings (confidence networks) with thickness of edges representing confidence in direct interaction between network nodes. Enrichment function provided was then used to test for enrichment of GO terms (GO biological processes, using standard settings).

## Supporting Information

S1 Fig Mutagenesis strategy for construction of the MCMV-gLuc reporter virus (ΔIE2-GFP-gLuc-MCMV). The GFP gene and the gLuc expression cassette were amplified (PCR I) and fused together by splicing PCR (PCR II). The product containing a Kanamycin resistance gene was subsequently used to replace the *Ie2* gene in the viral BACmid pSM3fr, producing a virus in which the reporter genes are under direct transcriptional control by the viral major IE-enhancer/promoter (MIEP).
(TIF)

S2 Fig Comparison of viral IE-gene and reporter gene expression during MCMV-gLuc infection. Expression kinetics of the viral *Ie1* gene and the *gLuc* reporter gene were compared by SYBRgreen qPCR detecting the respective mRNAs. Primary MEFs and BMDMs were infected (MCMV-gLuc) and RNA was isolated at indicated time points post infection (uninfected, 2, 4, 6 and 24 hpi). The *Ie1* and *gLuc* reporter genes are not detectable in the uninfected sample; therefore an arbitrary Ct value of 36 was set as a reference point.
(TIF)

S3 Fig Differences in TLR expression between BMDM and RAW264.7. Expression levels of TLR3, TLR4 and TLR7 were compared between BMDMs (n = 3) and RAW264.7 cells (n = 2) by relative quantitative PCR.
(TIF)

S4 Fig Effects of TLR ligands on viral entry. A) BMDMs were pre-treated with indicated TLR ligands for 15 min and subsequently infected (MCMV-gLuc). After adsorption cells were washed with medium and incubated for 6 h. At 6 hpi cells of three cultures were washed 3x with medium, trypsinised for 5 min and scraped off the culture plates for lysis and DNA isolation. Numbers of intra-cellular viral genomes were measured by absolute qPCR (n = 3, SE). B) Comparison of effectiveness of treatment to remove extracellular virus. BMDMs (n = 4) were infected (MOI 0.5) as described in S4 A Fig and either washed with PBS alone or additionally treated with either trypsin or citric acid to remove extracellular virus. DNA was subsequently isolated and M115 copies measured by qPCR.
(TIF)

S5 Fig TLR-agonists boost *Ie1* expression independent of tegument. A) RAW264.7 cells (24-well) were either mock treated or incubated with Pam3CSK4 (15 min) and subsequently infected with MCMV-ΔM45 (MOI = 1). *Ie1* expression was measured at 4 hpi (n = 3, mean fold change shown with SEM).
(TIF)

S6 Fig String network for siRNA targets >50% of max. knock-down. The list of all target genes that were identified in the siRNA screen to have >50% of the maximum knockdown effect (medium stringency) was used to produce an interaction network using the STRING online tool.
(TIF)

S7 Fig String network for siRNA targets >25% of max. knock-down. The list of all target genes that were identified in the siRNA screen to have >25% of the maximum knockdown effect (low stringency) was used to produce an interaction network using the STRING online tool.
(TIF)

S8 Fig Comparison of Ie1 levels between hMCMV and hMCMV-ΔRARE. BMDMs were infected (MOI = 1) with either hMCMV or hMCMV-ΔRARE and total RNA was harvested 4h p.i. and *Ie1* levels were measured by qPCR (n = 2).
(TIF)

S9 Fig Functional test of siRNA knockdown for IRFs and inducibility of IRF5 expression. A) MEFs were transfected either with control siRNA (RISC) or siRNAs targeting IRFs 1, 3, 5 or 7. Cells were subsequently infected with MCMV-gLuc and expression levels of IRF mRNAs were measured by relative qPCR and normalised to mock expression levels. B) IRF5 expression is inducible in RAW264.7 cells. IRF5 expression was measured by relative qPCR in infected cells (MCMV-gLuc) relative to mock samples.
(TIF)

S10 Fig ChIP experiments. A) RAW264.7 cells were infected with MCMV (MOI 0.5, 24 hpi) and used for ChIP analysis, using antibodies for IRFs 1, 3, 5 and 7 for pull-downs in comparison to unspecific IgG. Pull-downs for NFκB and Pol II were used as positive controls. Enrichment of host gene (*ActB*) or viral gene (*Ie1*) sequences was detected by SYBR-green qPCR. B) Summary of all ChIP experiments (n = 3) irrespective of experimental conditions for sAB IRF3, IRF5, p65 and Pol ll. Data points show fold enrichment over IgG control for individual experiments.
(TIF)

S1 Table Ranked lists of siRNA targets. List of siRNA targets sorted by their relative knockdown efficiency used to produce the STRING network graphs with the corresponding accession numbers. The three groups of stringency levels are indicated correspondingly.
(PDF)

S2 Table Relative gLuc activities and knock-down effects. Relative gLuc-activities for all siRNA targets and controls relative to the RISCfree control siRNA and the corresponding percentage of their knockdown effects relative to the maximum effect of the *gaussia luciferase* control siRNA.
(PDF)

S3 Table Ranked median activities of all siRNA targets for gLuc and GFP reporter assays. Ranked lists of all siRNA targets for GFP and gLuc reporter assays (compare to Fig. 5).
(PDF)

S4 Table Distribution of Replicates per siRNA screen. List of replicates distribution per siRNA screen used for statistical Meta-analysis.
(PDF)

S5 Table Sequence and position of NFκB/AP1/ATF binding motifs and point mutations. Positions of binding motifs in the human CMV enhancer are given relative to transcription start site of *Ie1*. Point mutations are shown and where applicable introduced endonuclease restriction sites are indicated by name of the enzyme.
(PDF)
