# Supplementary figures and images for: A Temporal Gate for Viral Enhancers to Co-opt Toll-Like-Receptor Transcriptional Activation Pathways upon Acute Infection
Source: PLoS Pathog. 2015 Apr 9;11(4):e1004737. doi: 10.1371/journal.ppat.1004737 (PMC4391941; doi:10.1371/journal.ppat.1004737)

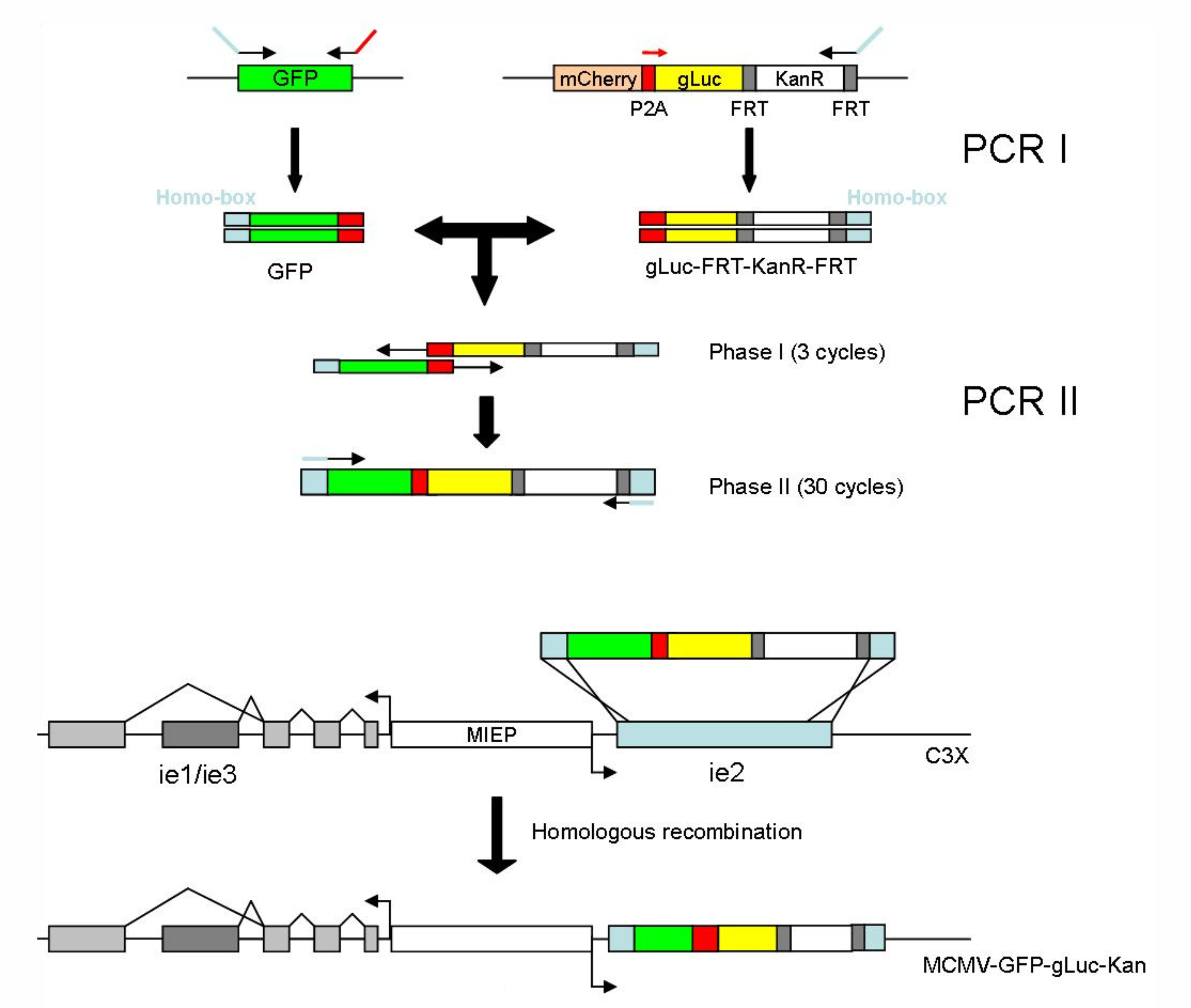

Supplement: S1 Fig — The GFP gene and the gLuc expression cassette were amplified (PCR I) and fused together by splicing PCR (PCR II). The product containing a Kanamycin resistance gene was subsequently used to replace the Ie2 gene in the viral BACmid pSM3fr, producing a virus in which the reporter genes are under direct transcriptional control by the viral major IE-enhancer/promoter (MIEP). (TIF) [file ppat.1004737.s001.tif]

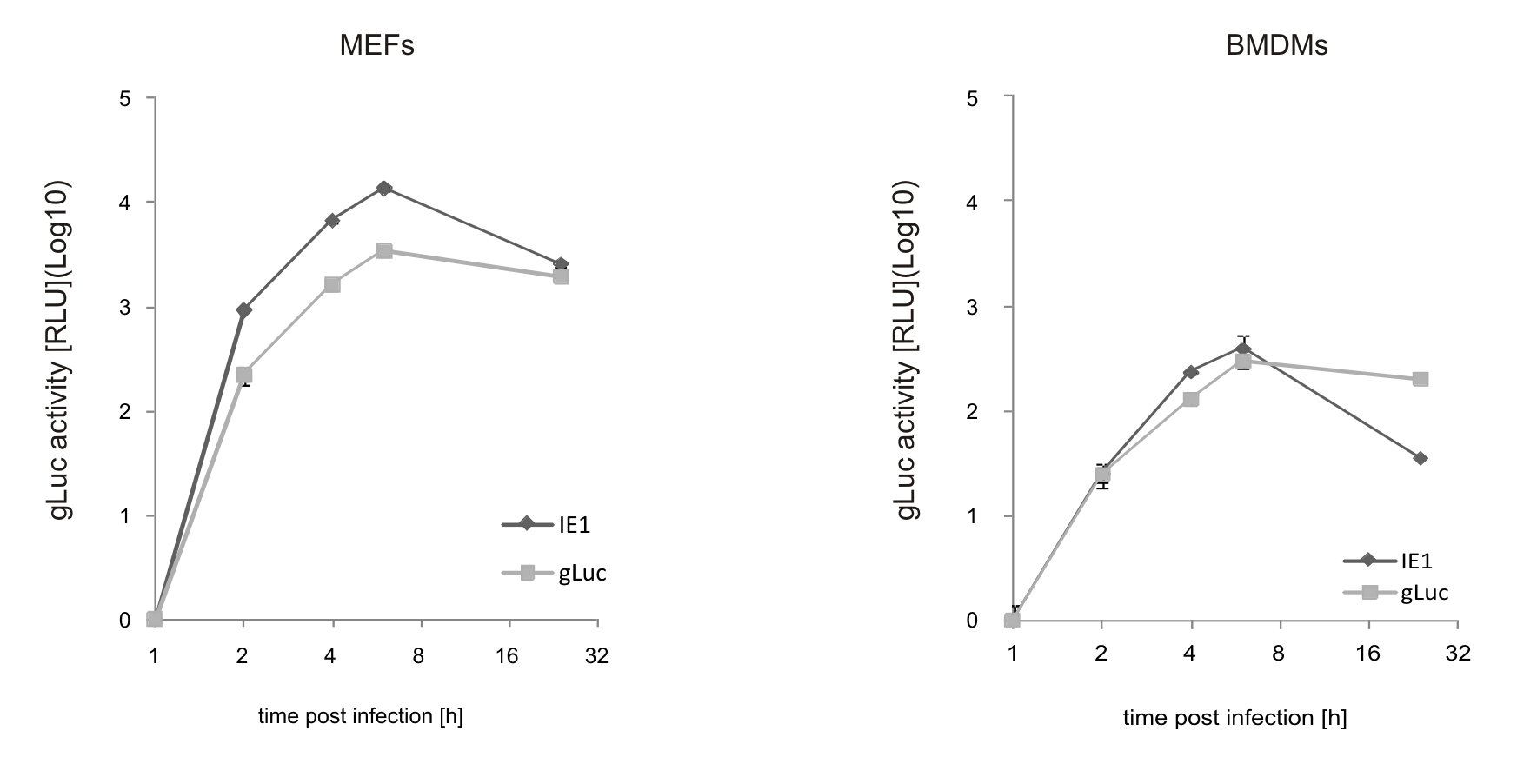

Supplement: S2 Fig — Expression kinetics of the viral Ie1 gene and the gLuc reporter gene were compared by SYBRgreen qPCR detecting the respective mRNAs. Primary MEFs and BMDMs were infected (MCMV-gLuc) and RNA was isolated at indicated time points post infection (uninfected, 2, 4, 6 and 24 hpi). The Ie1 and gLuc reporter genes are not detectable in the uninfected sample; therefore an arbitrary Ct value of 36 was set as a reference point. (TIF) [file ppat.1004737.s002.tif]

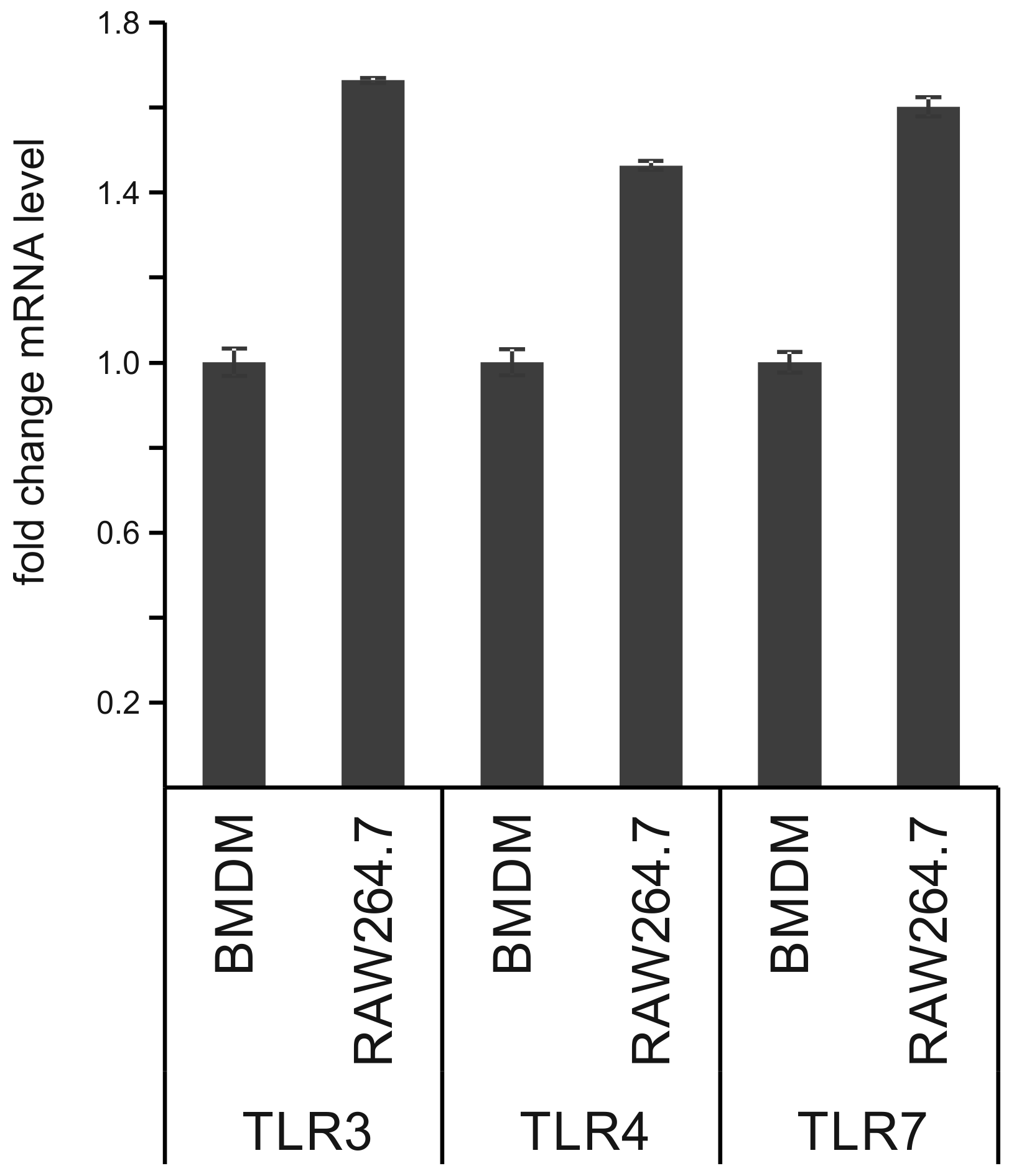

Supplement: S3 Fig — Expression levels of TLR3, TLR4 and TLR7 were compared between BMDMs (n = 3) and RAW264.7 cells (n = 2) by relative quantitative PCR. (TIF) [file ppat.1004737.s003.tif]

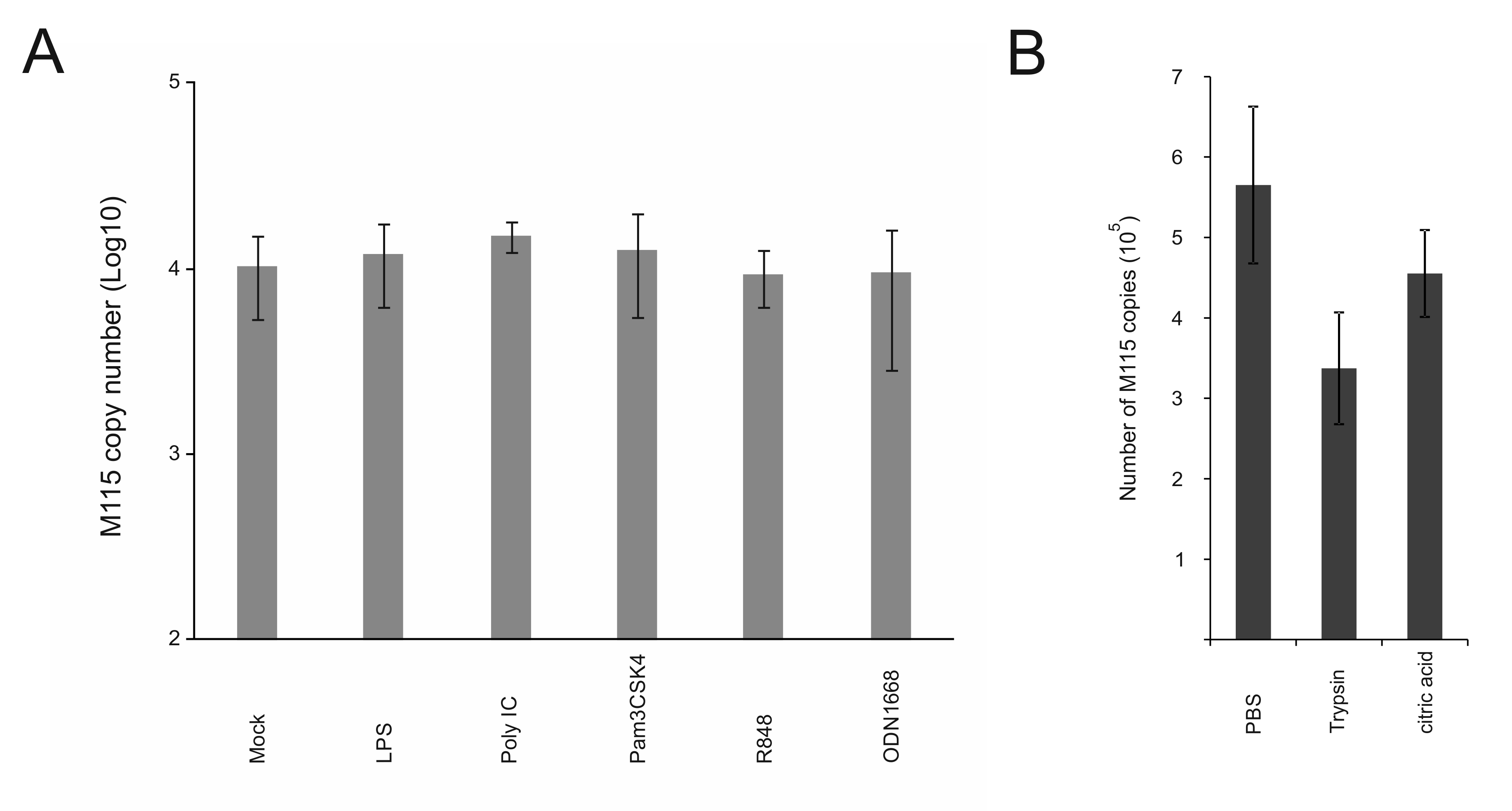

Supplement: S4 Fig — A) BMDMs were pre-treated with indicated TLR ligands for 15 min and subsequently infected (MCMV-gLuc). After adsorption cells were washed with medium and incubated for 6 h. At 6 hpi cells of three cultures were washed 3x with medium, trypsinised for 5 min and scraped off the culture plates for lysis and DNA isolation. Numbers of intra-cellular viral genomes were measured by absolute qPCR (n = 3, SE). B) Comparison of effectiveness of treatment to remove extracellular virus. BMDMs (n = 4) were infected (MOI 0.5) as described in S4 A Fig and either washed with PBS alone or additionally treated with either trypsin or citric acid to remove extracellular virus. DNA was subsequently isolated and M115 copies measured by qPCR. (TIF) [file ppat.1004737.s004.tif]

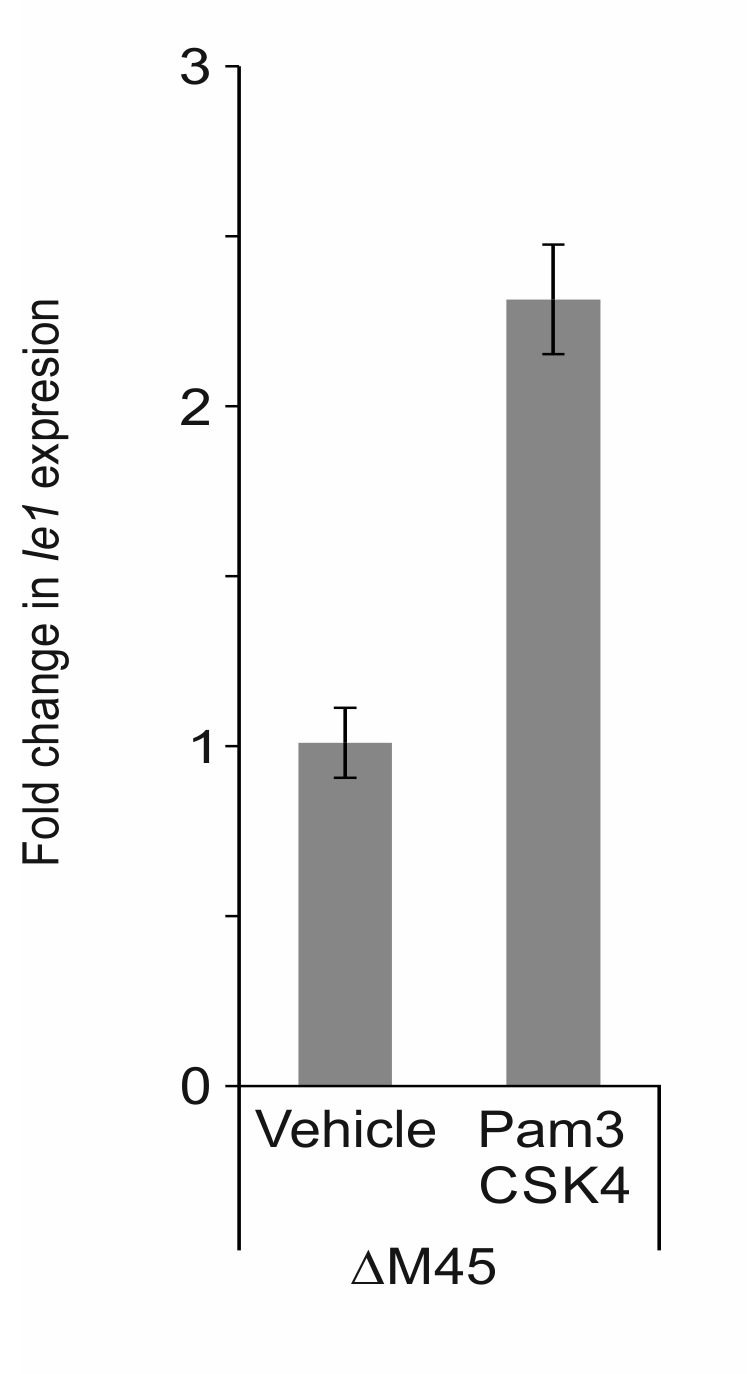

Supplement: S5 Fig — A) RAW264.7 cells (24-well) were either mock treated or incubated with Pam3CSK4 (15 min) and subsequently infected with MCMV-ΔM45 (MOI = 1). Ie1 expression was measured at 4 hpi (n = 3, mean fold change shown with SEM). (TIF) [file ppat.1004737.s005.tif]

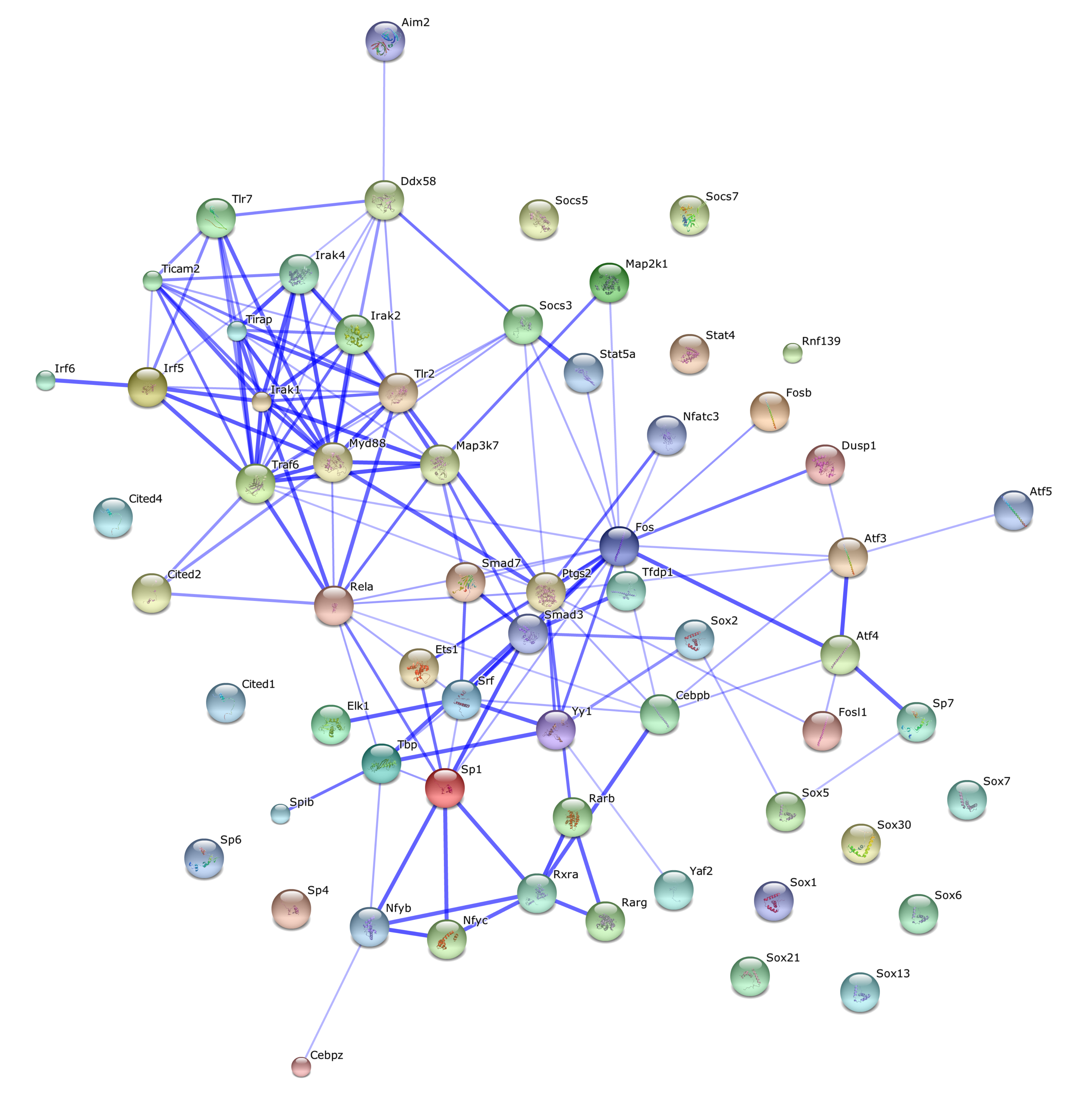

Supplement: S6 Fig — The list of all target genes that were identified in the siRNA screen to have >50% of the maximum knockdown effect (medium stringency) was used to produce an interaction network using the STRING online tool. (TIF) [file ppat.1004737.s006.tif]

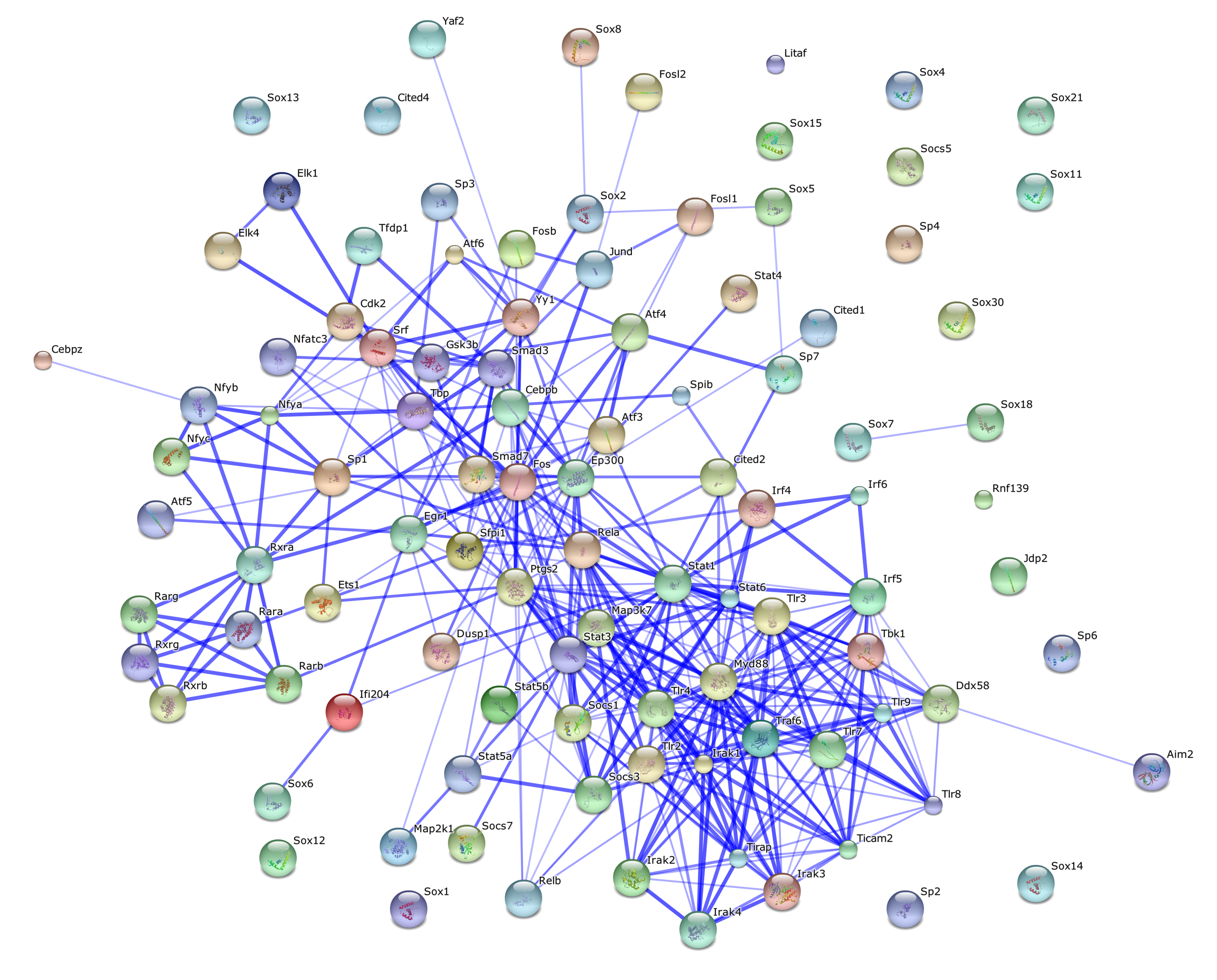

Supplement: S7 Fig — The list of all target genes that were identified in the siRNA screen to have >25% of the maximum knockdown effect (low stringency) was used to produce an interaction network using the STRING online tool. (TIF) [file ppat.1004737.s007.tif]

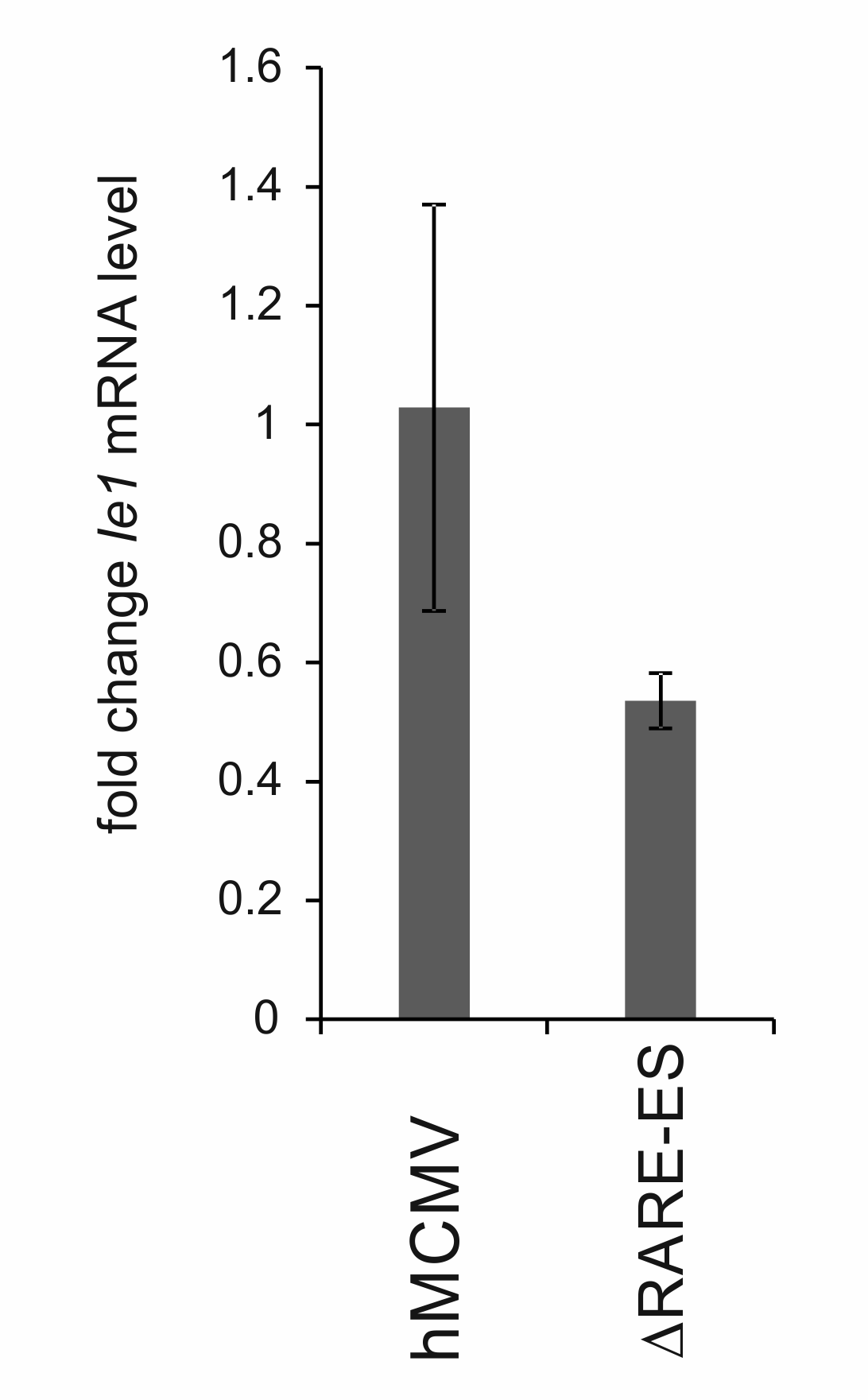

Supplement: S8 Fig — BMDMs were infected (MOI = 1) with either hMCMV or hMCMV-ΔRARE and total RNA was harvested 4h p.i. and Ie1 levels were measured by qPCR (n = 2). (TIF) [file ppat.1004737.s008.tif]

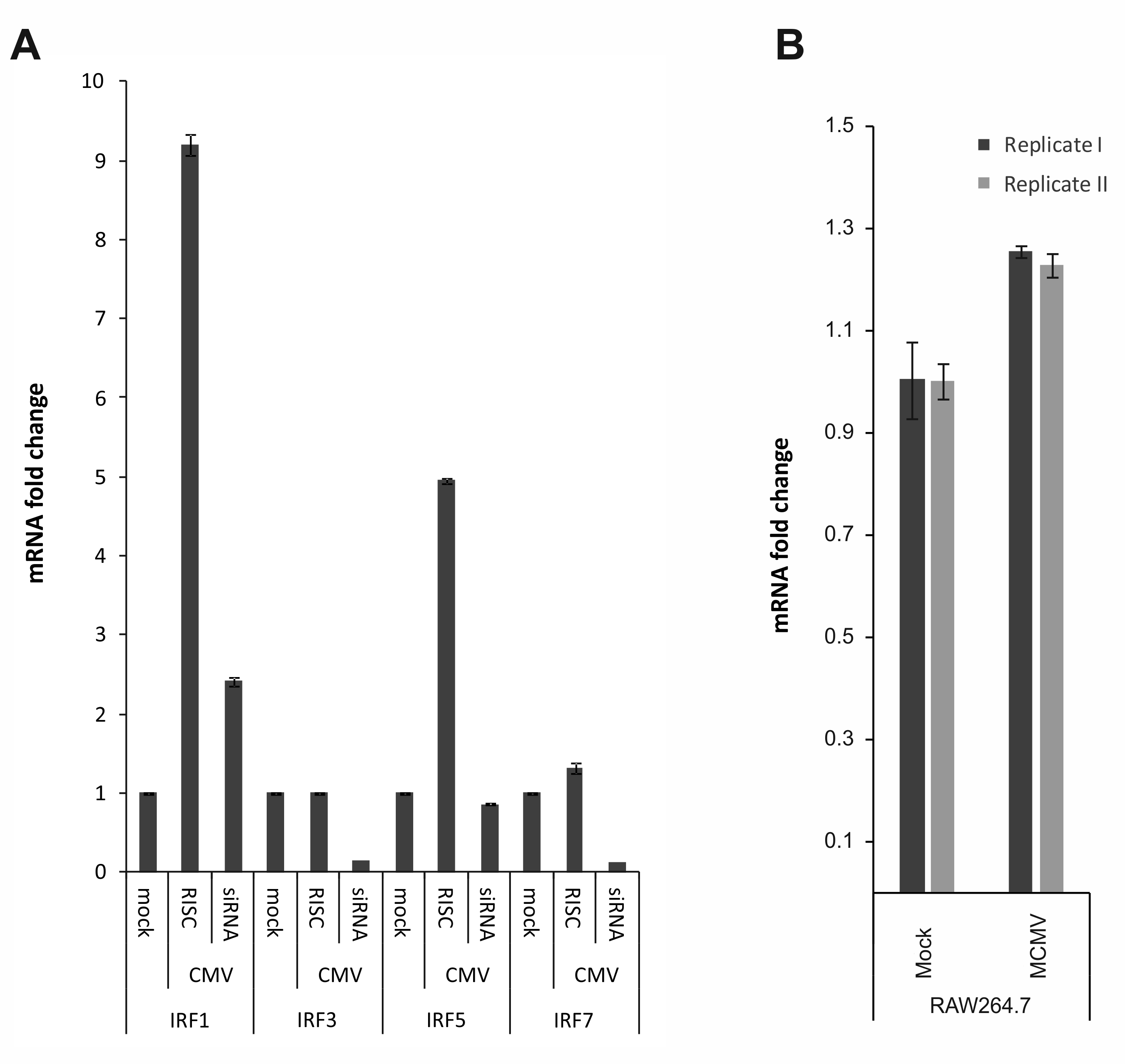

Supplement: S9 Fig — A) MEFs were transfected either with control siRNA (RISC) or siRNAs targeting IRFs 1, 3, 5 or 7. Cells were subsequently infected with MCMV-gLuc and expression levels of IRF mRNAs were measured by relative qPCR and normalised to mock expression levels. B) IRF5 expression is inducible in RAW264.7 cells. IRF5 expression was measured by relative qPCR in infected cells (MCMV-gLuc) relative to mock samples. (TIF) [file ppat.1004737.s009.tif]

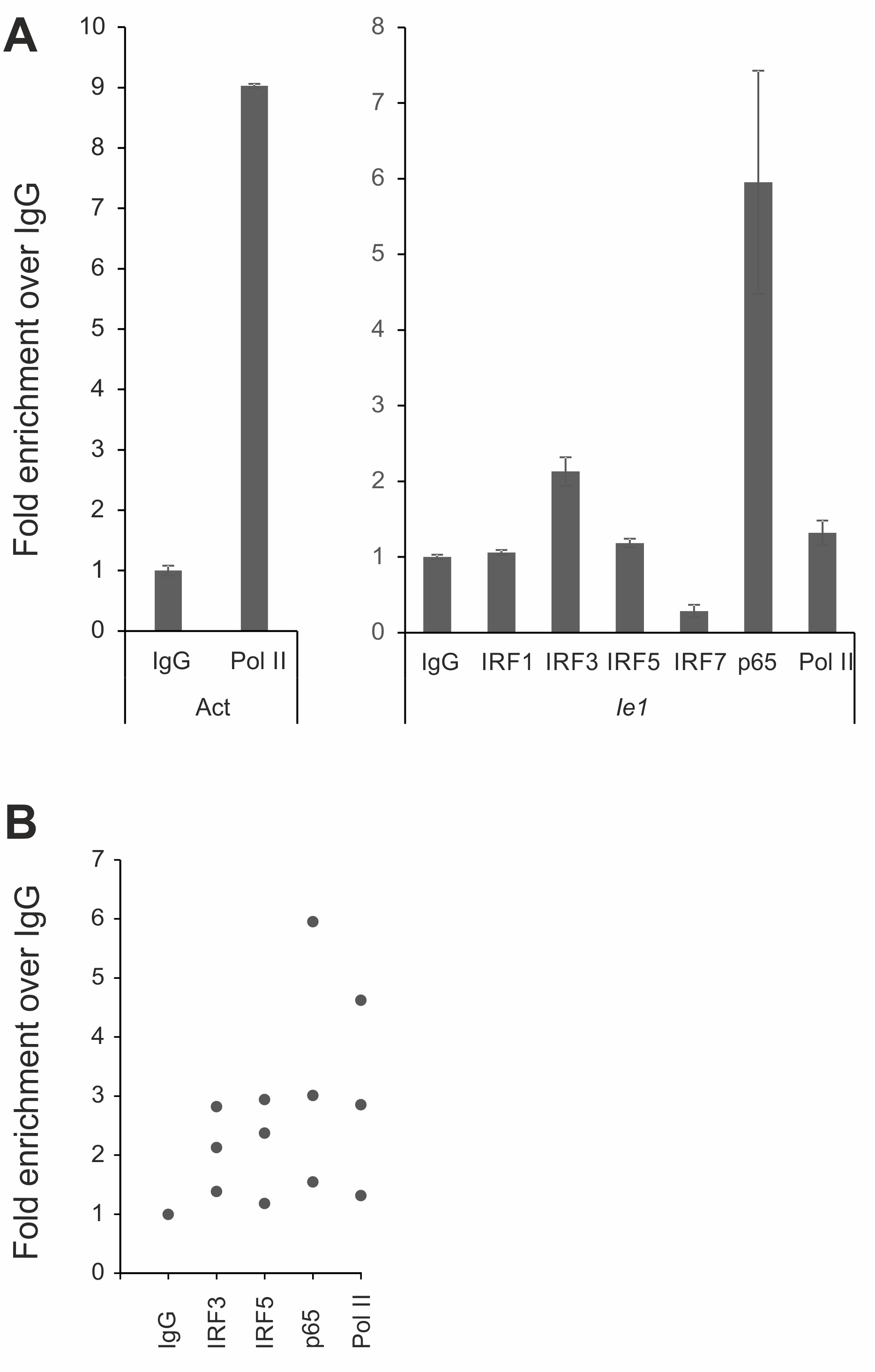

Supplement: S10 Fig — A) RAW264.7 cells were infected with MCMV (MOI 0.5, 24 hpi) and used for ChIP analysis, using antibodies for IRFs 1, 3, 5 and 7 for pull-downs in comparison to unspecific IgG. Pull-downs for NFκB and Pol II were used as positive controls. Enrichment of host gene (ActB) or viral gene (Ie1) sequences was detected by SYBR-green qPCR. B) Summary of all ChIP experiments (n = 3) irrespective of experimental conditions for sAB IRF3, IRF5, p65 and Pol ll. Data points show fold enrichment over IgG control for individual experiments. (TIF) [file ppat.1004737.s010.tif]
