# Supplementary material for: A Temporal Gate for Viral Enhancers to Co-opt Toll-Like-Receptor Transcriptional Activation Pathways upon Acute Infection
Source: PLoS Pathog. 2015 Apr 9;11(4):e1004737. doi: 10.1371/journal.ppat.1004737 (PMC4391941; doi:10.1371/journal.ppat.1004737)
Supplement: S1 Table — List of siRNA targets sorted by their relative knockdown efficiency used to produce the STRING network graphs with the corresponding accession numbers. The three groups of stringency levels are indicated correspondingly. (PDF) [file ppat.1004737.s011.pdf]

	>75% max KD	
Rank	Gene Name	Accession #
1	Sp1	NM_013672
2	Nfyb	NM_010914.2
3	RXRA	NM_011305
4	SOX13	NM_011439.2
5	IRAK4	NM_029926.5
6	AIM2	NM_001013779.2
7	DDX58	NM_172689.3
8	TLR7	NM_133211.3
9	ATF4	NM_009716
10	SOCS3	NM_007707
11	IRAK1	NM_001177973.1
12	SMAD7	NM_001042660.1
13	IRF5	NM_012057
14	SOCS7	NM_138657
15	SP4	NM_009239
16	YAF2	NM_024189
17	CITED1	NM_007709
18	TIRAP	NM_001177845.1
19	Nfyg	NM_001048168.1
20	RELA	NM_009045
21	MyD88	NM_010851.2
22	Ets-1	NM_001038642.1
23	SOX1	NM_009233
24	CITED2	NM_010828
25	YY1	NM_009537

	>50% max KD	
Rank	Gene Name	Accession #
26	TICAM2 (TRAM)	NM_173394.3
27	SRF	NM_020493
28	MKP-1 (DUSP1)	NM_013642
29	SOCS5	NM_019654
30	PTGS2 (Cox2)	NM_011198.3
31	TFDp1/Dp1/DrTF1	NM_009361
32	ATF3	NM_007498
33	SMAD3	NM_016769.4
34	MEKK1	NM_011945
35	FOS	NM_010234
36	CEBPb	NM_024125.4
37	NFAT4(NFATc3)	NM_010901.2
38	SOX2	NM_011443.3
39	IRAK-2	NM_001113553.1
40	RNF139	NM_175226
41	SOX21	NM_177753.3
42	STAT5A	NM_011488
43	TLR-2	NM_011905.3
44	SOX30	NM_173384.2
45	SP6	NM_031183
46	SOX7	NM_011446.1
47	FOSB	NM_008036
48	SPI-B	NM_019866.1
49	TBP	NM_013684
50	SP7	NM_130458
51	ELK1	NM_007922
52	RARG	NM_001042727
53	TAK-1 (Map3k7)	NM_009316.1
54	CITED4	NM_019563
55	TRAF6	NM_009424.2
56	SOX5	NM_001113559.1
57	CEBPz	NM_001024806.2
58	RARB	NM_011243
59	IRF6	NM_016851
60	SOX6	NM_001025560.1
61	Fosl1	NM_010235
62	ATF5	NM_030693
63	Stat4	NM_011487

	>25% max KD			<25% max KD	
Rank	Gene Name	Accession #	Rank	Gene Name	Accession #
64	SOX12	NM_011438.2	107	CREB3L1	NM_011957
65	SOX15	NM_009235.1	108	CREBL1	NM_017406
66	GSK3b	NM_019827.6	109	SARM1	NM_001168521.1
67	TLR-9	NM_031178.2	110	IRF9	NM_001159417.1
68	IRF4	NM_013674	111	SOCS6	NM_018821
69	TLR-8	NM_133212.2	112	IRF3	NM_016849
70	IFI16 (ifi204)	NM_008329.2	113	SOX3	NM_009237.2
71	ELK4	NM_007923	114	CREB3	NM_013497
72	RXRG	NM_009107	115	E2F1	NM_007891
73	SP3	NM_001098425	116	NFkBIB	NM_010908
74	RXRB	NM_011306	117	CdK1	NM_007659
75	JUND1	NM_010592	118	IRF7	NM_016850
76	TBK-1	NM_019786.4	119	MDA-5 (IFih1)	NM_001164477.1
77	SOCS1	NM_009896	120	IRF2	NM_008391
78	EP300	NM_177821	121	ZBP-1	NM_001139519.1
79	RARA	NM_009024	122	CEBPg	NM_009884.3
80	SOX14	NM_011440.1	123	IRF1	NM_008390
81	TLR-4	NM_021297.2	124	IRF8	NM_008320.3
82	Pu.1 (SPI-1)	NM_011355.1	125	SOCS4	NM_080843
83	SOX4	NM_009238.2	126	SOX9	NM_011448.4
84	TLR-3	NM_126166.4	127	NFKB2	NM_019408
85	RELB	NM_009046	128	NFAT3(NFATc4)	NM_023699.3
86	CdK2	NM_183417	129	JUNB	NM_008416
87	SOX11	NM_009234.6	130	CREB3L4	NM_030080
88	FOSL2	NM_008037	131	CREB1	NM_001037726
89	STAT3	NM_011486	132	ATF2	NM_009715
90	SOX18	NM_009236.2	133	REL	NM_009044
91	EGR-1	NM_007913.5	134	SOX17	NM_011441.4
92	SOX8	NM_011447.3	135	STAT2	NM_019963
93	STAT1	NM_009283	136	SOX10	NM_011437
94	IRAK-3	NM_028679.3	137	NFKB1	NM_008689
95	ATF6	NM_001081304	138	ATF1	NM_007497
96	Nfy-alpha	NM_001110832.1	139	TP53	NM_011640.3
97	LITAF(PIG7)	NM_019980.2	140	SOCS2	NM_001168657
98	SP2	NM_001080964	141	NFATc(NFATc1)	NM_001164109.1
99	JUNDM2	NM_030887	142	CREBBP	NM_001025432
100	Stat6	NM_009284	143	JUN	NM_010591
101	STAT5B	NM_001113563	144	EGR-3	NM_018781.2
102	ELK3	NM_013508	145	CEBPa	NM_007678.3
103	TICAM1 (TRIF)	NM_174989.4	146	NFAT5(TonEBP)	NM_133957.3
104	Ets-2	NM_011809.3	147	NFATp(NFATc2)	NM_001037177.1
105	Pik3ca	NM_008839	148	EGR-2	NM_010118.3
106	CIP21	NM_007669	149	ATF7	NM_146065
