# Supplementary material for: A Temporal Gate for Viral Enhancers to Co-opt Toll-Like-Receptor Transcriptional Activation Pathways upon Acute Infection
Source: PLoS Pathog. 2015 Apr 9;11(4):e1004737. doi: 10.1371/journal.ppat.1004737 (PMC4391941; doi:10.1371/journal.ppat.1004737)
Supplement: S2 Table — Relative gLuc-activities for all siRNA targets and controls relative to the RISCfree control siRNA and the corresponding percentage of their knockdown effects relative to the maximum effect of the gaussia luciferase control siRNA. (PDF) [file ppat.1004737.s012.pdf]

siRNA target	average of gLuc activity (relative to RISC)	SE	knock-down relative to gLuc siRNA [%]
AIM2	0.528536191	0.072602693	98.87462857
ATF1	1.085875823	0.049352847	-18.00973898
ATF2	1.059620806	0.054264148	-12.50357914
ATF3	0.697888009	0.05278688	63.35843885
ATF4	0.550981407	0.101981909	94.16745414
ATF5	0.760259786	0.038899933	50.27793041
ATF6	0.851377285	0.014627544	31.16891573
ATF7	1.536323179	0.073735658	-112.476831
CdK1	0.960568989	0.034623944	8.269407873
CdK2	0.827407589	0.032231917	36.19580168
CEBPa	1.210468779	0.643350823	-44.13917251
CEBPb	0.705330475	0.35253884	61.79761693
CEBPg	1.017522358	0.275329844	-3.67476058
CEBPz	0.743103866	0.225711305	53.87584239
CIP21	0.912845227	0.108972456	18.27795833
CITED1	0.605702118	0.049058589	82.69151504
CITED2	0.641380857	0.023073191	75.20902746
CITED4	0.740580744	0.037955334	54.4049875
CREB1	1.056847112	0.075518015	-11.9218845
CREB3	0.950414487	0.07539841	10.39899369
CREB3L1	0.923532574	0.091801907	16.03662512
CREB3L4	1.047519766	0.088193606	-9.965768569
CREBBP	1.142880572	0.087835987	-29.96468277
CREBL1	0.940585567	0.087450087	12.46029903
E2F1	0.95198109	0.060686282	10.07044817
EGR-1	0.832581253	0.097535946	35.1107892
EGR-2	1.42011632	0.115721298	-88.10611606
EGR-3	1.193567786	0.156613629	-40.59472352
ELK1	0.737806474	0.067353879	54.98680276
ELK3	0.882199993	0.078311069	24.70482726
ELK4	0.786362192	0.089886445	44.80377612
EP300	0.807858141	0.07680431	40.29568028
Ets-1	0.639168694	0.091681015	75.67295885
Ets-2	0.895592489	0.074794155	21.89617466
FOS	0.701870819	0.048559029	62.52317028
FOSB	0.72751551	0.068354308	57.14500727
Fosl1	0.758419377	0.034559776	50.66389822
FOSL2	0.82947012	0.049649749	35.7632511
gaussia Luciferase	0.523170084	0.043207968	100
GFP	1.043640059	0.064305726	-9.152122709
GSK3b	0.768758388	0.100269407	48.49561733
IFI16 (ifi204)	0.780777329	0.092331292	45.97502447
IRAK-1	0.557149208	0.085736777	92.87395286
IRAK-2	0.708392471	0.085976694	61.15546013
IRAK-3	0.844392168	0.133901202	32.63382321
IRAK-4	0.519499499	0.043126185	100.7697892
IRF1	1.018301276	0.221352398	-3.838113978
IRF2	1.000471068	0.244714184	-0.098791657
IRF3	0.947990863	0.28798701	10.90727213
IRF4	0.773673415	0.133715156	47.46484589
IRF5	0.596686977	0.093472245	84.5821558

IRF6	0.74841723	0.092792706	52.76153223
IRF7	0.985496698	0.049899813	3.041609059
IRF8	1.018657514	0.105541267	-3.912823721
IRF9	0.945526097	0.079045823	11.42417888
JUN	1.158650108	0.035338198	-33.27184446
JUNB	1.036601925	0.05716168	-7.67609657
JUND1	0.797564733	0.14861131	42.45439718
JUNDM2	0.863475739	0.040696579	28.63164762
LITAF(PIG7)	0.860970109	0.150787342	29.15712421
M54 (viral Polymerase)	0.906512575	0.023582464	19.60603173
MDA-5 (IFih1)	0.987407983	0.098767828	2.640777406
MEKK1	0.701598217	0.127433242	62.58033994
MKP-1 (DUSP1)	0.679975303	0.018619929	67.11506264
MyD88	0.635096092	0.095573854	76.52705825
NFAT3(NFATc4)	1.035119675	0.105165497	-7.365241432
NFAT4(NFATc3)	0.705724039	0.166742597	61.71507935
NFAT5(TonEBP)	1.21820964	0.129621565	-45.7625733
NFATc(NFATc1)	1.115875914	0.127499022	-24.30130963
NFATp(NFATc2)	1.38292476	0.187214149	-80.30636226
NFKB1	1.085495882	0.095381587	-17.93005833
NFKB2	1.032508583	0.123669376	-6.817647473
NFkBIB	0.958543881	0.097581939	8.694110223
Nfy-alpha	0.860624564	0.112408202	29.22959135
Nfy-beta	0.481189526	0.057374024	108.8040948
Nfy-gamma	0.62036817	0.0979905	79.61577422
non Targenting pool	0.943069322	0.109488068	11.93940992
Pik3ca	0.904646488	0.071367089	19.99738464
PTGS2 (Cox2)	0.685561614	0.080317796	65.94351046
Pu.1 (SPI-1)	0.818257896	0.234300881	38.11466065
RARA	0.812421657	0.051396008	39.33862715
RARB	0.746963444	0.06435032	53.06641793
RARG	0.738173521	0.078048151	54.90982632
RISC	0.935198582	0.0814679	13.59004871
RISC	1.058010905	0.146629679	-1.424095434
RISC	1.006790513	0.051216058	-12.16595328
REL	1.063573164	0.065940474	-13.33246137
RELA	0.622779867	0.092255186	79.10999714
RELB	0.82335095	0.047023715	37.04655358
RIG-I (DDX58)	0.541696721	0.04210055	96.1146235
RNF139	0.70860201	0.0395074	61.11151596
RXRA	0.483741624	0.081525602	108.2688729
RXRB	0.794795864	0.050410048	43.03507998
RXRG	0.787727549	0.041057674	44.51743568
SARM1	0.943548205	0.114210159	11.83897937
SMAD3	0.701037323	0.120480488	62.69796986
SMAD7	0.570706076	0.077786397	90.03082851
SOCS1	0.802312404	0.087521844	41.45872343
SOCS2	1.113175821	0.09144381	-23.73505044
SOCS3	0.553153725	0.016186465	93.71187913
SOCS4	1.030184869	0.132934127	-6.330321937
SOCS5	0.684113259	0.048789127	66.24725715
SOCS6	0.94579435	0.107441063	11.3679213
SOCS7	0.602367978	0.059706952	83.39074559

SOX1	0.63961034	0.072610306	75.58033745
SOX10	1.084982863	0.165183387	-17.82246869
SOX11	0.829343589	0.112584422	35.78978698
SOX12	0.767830451	0.145365221	48.69022289
SOX13	0.515819159	0.101122865	101.5416241
SOX14	0.812956261	0.096511033	39.22651092
SOX15	0.768262261	0.102875331	48.5996644
SOX17	1.068358548	0.146453152	-14.33604437
SOX18	0.831030006	0.098239984	35.43611418
SOX2	0.706899038	0.083733955	61.46866047
SOX21	0.714055614	0.11569316	59.96779478
SOX3	0.949872485	0.104033767	10.51266158
SOX30	0.722655004	0.144018451	58.16434472
SOX4	0.819208813	0.052153232	37.91523568
SOX5	0.740875721	0.072951599	54.34312536
SOX6	0.757644873	0.066618379	50.82632588
SOX7	0.727476704	0.046292947	57.1531455
SOX8	0.840476475	0.124413251	33.45501597
SOX9	1.031022429	0.132655469	-6.505973729
Sp1	0.479386339	0.039252644	109.1822563
SP2	0.863284562	0.050154852	28.67174079
SP3	0.787835803	0.023639764	44.49473275
SP4	0.603952127	0.066524414	83.05852029
SP6	0.726712153	0.033832103	57.31348591
SP7	0.737608346	0.102940626	55.02835389
SPI-B	0.73059214	0.153419884	56.49978126
SRF	0.673382432	0.049341096	68.49770882
STAT1	0.840806372	0.028559131	33.38583068
STAT2	1.069354507	0.059354828	-14.54491525
STAT3	0.831000412	0.057109919	35.4423207
STAT4	0.76144664	0.089873447	50.02902535
STAT5A	0.714813726	0.032651227	59.80880479
STAT5B	0.877635721	0.055905339	25.66203895
STAT6	0.870406859	0.053111464	27.17806425
TAK-1 (Map3k7)	0.739489957	0.079739163	54.63374544
TBK-1	0.802118831	0.041215951	41.49931912
TBP	0.735396388	0.075121968	55.49224215
TFDp1 (Dp1)	0.691655011	0.030606834	64.66561323
TICAM1 (TRIF)	0.889081049	0.084266825	23.2617432
TICAM2 (TRAM)	0.663029739	0.10578713	70.66885905
TIRAP (Mal)	0.606195712	0.036214675	82.58799937
TLR-2	0.715276751	0.088238964	59.71169998
TLR-3	0.822593381	0.11167837	37.20542962
TLR-4	0.816700206	0.097703674	38.44133683
TLR-7	0.5467444	0.089393704	95.05603254
TLR-8	0.774118913	0.080512875	47.37141676
TLR-9	0.770027666	0.139008566	48.2294265
TP53	1.10305556	0.103180392	-21.61264552
TRAF6	0.740691365	0.113111245	54.38178812
YAF2	0.603956487	0.046538024	83.05760604
YY1	0.642444071	0.042368252	74.98605199
ZBP-1	1.008306212	0.142208451	-1.741965338
