# Supplementary material for: A Temporal Gate for Viral Enhancers to Co-opt Toll-Like-Receptor Transcriptional Activation Pathways upon Acute Infection
Source: PLoS Pathog. 2015 Apr 9;11(4):e1004737. doi: 10.1371/journal.ppat.1004737 (PMC4391941; doi:10.1371/journal.ppat.1004737)
Supplement: S3 Table — Ranked lists of all siRNA targets for GFP and gLuc reporter assays (compare to Fig. 5). (PDF) [file ppat.1004737.s013.pdf]

GFP-replication assay	siglabels *=significant	sirna.median	sirna.sem	p-value (wilcoxon signed rank test)
M86	M86 *	-4.022897366	0.727715929	0.0078125
M54	M54 *	-3.481211778	1.162503798	0.0078125
SOX21	SOX21 *	-2.208443084	0.947683881	0.009765625
TFDp1/Dp1/DrTF1	TFDp1/Dp1/DrTF1	-2.070481805	0.346274039	0.125
CdK1	CdK1	-2.000484692	0.967830356	0.25
SOX7	SOX7 *	-1.954044884	0.597168002	0.001953125
CEBPz	CEBPz *	-1.903657017	0.408929306	0.001953125
TBP	TBP *	-1.776582716	0.558074116	0.001930237
SREBf2	SREBf2	-1.751344583	1.680244524	0.15625
RXRA	RXRA *	-1.681121141	0.708409713	0.000328064
MEKK1	MEKK1	-1.653921833	0.987097625	0.375
Pu.1 (SPI-1)	Pu.1 (SPI-1) *	-1.607245571	0.461678742	0.00390625
Scap	Scap	-1.564218472	2.091818366	0.0625
Sp1	Sp1 *	-1.437726207	0.34610472	0.000144958
SOX5	SOX5 *	-1.429055674	0.548502931	0.00390625
LITAF(PIG7)	LITAF(PIG7)	-1.404283144	0.598985565	0.064453125
Fosl1	Fosl1 *	-1.224653743	0.313627395	7.63E-06
Nfy-gamma	Nfy-gamma	-1.21760429	0.409137033	0.064453125
RXRG	RXRG *	-1.169301841	0.230303615	7.63E-06
ATF6	ATF6 *	-1.148916547	0.494125055	0.000534058
gaussia Luciferase	gaussia Luciferase *	-1.097394089	0.152383086	0.001289368
YY1	YY1 *	-1.093750456	0.468370829	0.00769043
IE3	IE3 *	-1.074641865	0.283830999	0.000488281
SP4	SP4 *	-1.059988979	0.395866566	0.000671387
Insig1	Insig1	-1.019509317	1.698649936	0.0625
CITED1	CITED1 *	-0.996325356	0.354316457	7.63E-06
FOSL2	FOSL2 *	-0.995782508	0.238980853	0.000839233
AIM2	AIM2 *	-0.991851471	0.46505839	0.001953125
NFkBIB	NFkBIB *	-0.990581354	0.713351477	0.01953125
TLR-3	TLR-3	-0.946068547	0.488085915	0.193359375
IRAK-1	IRAK-1	-0.945308285	0.629677214	0.16015625
EGR-1	EGR-1	-0.879698866	0.450717895	0.322265625
EP300	EP300 *	-0.878221315	0.379391659	1.53E-05
ELK1	ELK1 *	-0.86531322	0.258071618	0.008964539
ATF5	ATF5 *	-0.816889669	0.552338008	0.000534058
ATF4	ATF4 *	-0.811292758	0.235397756	0.034233093
NFAT4(NFATc3)	NFAT4(NFATc3) *	-0.800159764	0.376296122	0.048828125
SOX10	SOX10	-0.793606802	0.577197236	0.16015625
IRAK-3	IRAK-3	-0.791087036	0.296222918	0.10546875
CITED2	CITED2 *	-0.790435465	0.267015081	0.00769043
IRF6	IRF6 *	-0.7894504	0.118777497	0.004005432
RARB	RARB *	-0.767604363	0.226691887	0.010406494
FOS	FOS *	-0.762955215	0.272183563	0.000190735
JUN	JUN *	-0.742307128	0.178863819	0.001045227
SP7	SP7 *	-0.732161998	0.14483205	0.023674011
CEBPg	CEBPg *	-0.730104874	0.295478532	0.013671875
CdK2	CdK2	-0.707981507	0.741360978	0.875
JUND1	JUND1 *	-0.683585888	0.208287561	7.63E-06
FOSB	FOSB *	-0.649382997	0.199340088	0.001045227
NFKBIB	NFKBIB *	-0.648027275	0.106268576	0.0078125
IRAK-2	IRAK-2 *	-0.647564936	0.563746655	0.048828125
SOX18	SOX18 *	-0.632791525	0.243528132	0.048828125
ELK3	ELK3 *	-0.627907925	0.179978935	7.63E-06
TLR-9	TLR-9 *	-0.621278381	0.685066258	0.00390625
Nfy-beta	Nfy-beta	-0.59623455	0.711993851	0.845703125
STAT3	STAT3 *	-0.593153258	0.157217581	0.018234253
STAT5A	STAT5A *	-0.588401111	0.412433057	7.63E-06
IRF5	IRF5 *	-0.585769858	0.194413528	0.000144958
PTGS2 (Cox2)	PTGS2 (Cox2)	-0.578270308	0.347591316	0.322265625
CREB3L1	CREB3L1 *	-0.578268337	0.110124551	0.015930176
Stat4	Stat4	-0.576599067	0.237520461	0.228752136
RNF139	RNF139	-0.575806031	0.246259806	0.125
SRF	SRF *	-0.565224255	0.318954854	0.006576538
SOCS7	SOCS7	-0.548690757	0.521298863	0.375
CEBPb	CEBPb *	-0.531515566	0.568104692	0.001953125
NFKB2	NFKB2 *	-0.508470869	0.229686208	0.000839233

TIRAP (Mal)	TIRAP (Mal)	-0.502460839	0.457808218	0.375
CREB3	CREB3 *	-0.484306569	0.214712466	0.023674011
ZBP-1	ZBP-1	-0.476219737	0.214144221	0.10546875
JUNB	JUNB	-0.475006011	0.207196147	0.181465149
SP3	SP3 *	-0.465999948	0.165406837	0.038490295
GSK3b	GSK3b	-0.457545142	0.71061707	0.845703125
SOCS5	SOCS5	-0.452625389	0.187789192	0.125
MKP-1 (DUSP1)	MKP-1 (DUSP1)	-0.448993013	0.535931225	0.875
SPI-B	SPI-B	-0.436907622	0.448939683	0.275390625
TP53	TP53	-0.433098381	0.549190355	0.322265625
ATF7	ATF7 *	-0.416458183	0.160896506	0.013870239
IRF4	IRF4 *	-0.41220686	0.328089979	0.004745483
CITED4	CITED4 *	-0.411755198	0.175165648	0.000534058
ATF2	ATF2 *	-0.407532124	0.218462944	0.000839233
SARM1	SARM1	-0.403340771	0.342746732	0.322265625
RISC	RISC	-0.401289524	0.247726308	0.375
NFAT3(NFATc4)	NFAT3(NFATc4)	-0.401271906	0.835557368	0.845703125
SP6	SP6 *	-0.384230402	0.083289285	0.03036499
SOCS1	SOCS1	-0.381224068	0.421388815	0.875
RELA	RELA *	-0.374465011	0.300260293	0.012031555
non Targenting pool#1	non Targenting pool#1 *	-0.360748143	0.162700226	0.013870239
ATF1	ATF1	-0.349585186	0.140009797	0.283729553
BAG	BAG *	-0.347604189	0.134078428	0.0078125
CREBBP	CREBBP *	-0.34585353	0.222236771	0.026847839
JUNDM2	JUNDM2 *	-0.317651002	0.162853203	0.013870239
SOCS3	SOCS3	-0.309639267	0.35022347	0.625
TICAM1 (TRIF)	TICAM1 (TRIF)	-0.308105242	0.3254901	0.193359375
Stat6	Stat6 *	-0.297248744	0.229355345	0.020812988
RARG	RARG	-0.290066371	0.257007505	0.212142944
SOCS2	SOCS2	-0.282947029	0.106601712	0.125
E2F1	E2F1 *	-0.28005041	0.149357837	0.034233093
SOX2	SOX2	-0.271011659	0.28093086	0.556640625
IRF7	IRF7	-0.256820029	0.431910396	0.639694214
STAT5B	STAT5B *	-0.256442092	0.097661976	0.048278809
NFAT5(TonEBP)	NFAT5(TonEBP)	-0.254639852	0.467846531	0.4921875
SP2	SP2 *	-0.253633557	0.144349679	0.015930176
SOX13	SOX13	-0.247556386	0.49383115	1
YAF2	YAF2	-0.238708284	0.191168246	0.089767456
EGR-2	EGR-2	-0.23207364	0.728933775	0.921875
mock	mock *	-0.228901402	0.148552584	0.0390625
TLR-2	TLR-2	-0.22630508	0.428669592	0.921875
RISC	RISC *	-0.225195388	0.20530725	0.042489171
TRAF6	TRAF6 *	-0.223116598	0.491848133	0.048828125
CEBPa	CEBPa	-0.221469998	0.324565787	0.232421875
Nfy-alpha	Nfy-alpha	-0.220058657	0.460651885	0.375
IRF8	IRF8	-0.203061768	1.014356085	0.130859375
ELK4	ELK4	-0.20082445	0.107265461	0.167350769
REL	REL	-0.198117456	0.115751002	0.081428528
CREB1	CREB1	-0.18631907	0.211798261	0.228752136
SOCS6	SOCS6	-0.176449753	0.17103715	0.25
CIP21	CIP21	-0.160304372	0.554443382	0.875
RARA	RARA	-0.159797626	0.171780936	0.579841614
RXRB	RXRB	-0.14680579	0.068391072	0.053855896
NFKB1	NFKB1	-0.126189116	0.077450824	0.196388245
RELB	RELB	-0.116335813	0.408001856	0.966117859
Daxx	Daxx	-0.115641145	0.27772407	0.25
SMAD3	SMAD3	-0.114949006	1.03370964	1
CREBL1	CREBL1	-0.094639348	0.129522846	0.86504364
CREB3L4	CREB3L4	-0.089019449	0.124335557	0.495079041
SOX15	SOX15	-0.088526942	0.267158148	0.921875
IRAK-4	IRAK-4	-0.070019215	0.537414441	0.845703125
SOX4	SOX4	-0.064720524	0.315331594	0.76953125
STAT1	STAT1	-0.06322075	0.301494601	0.670524597
SOX11	SOX11	-0.061135261	0.620388945	1
ATF3	ATF3	-0.029086887	0.229315891	0.932281494
SOX12	SOX12	-0.027980511	0.570552497	0.845703125
SOX17	SOX17	-0.003042833	0.338773966	1

SOX6	SOX6	0.022125714	0.558350914	0.921875
MDA-5 (IFih1)	MDA-5 (IFih1)	0.042475943	0.518628643	0.921875
SOX30	SOX30	0.042572019	0.336799575	0.921875
IRF1	IRF1	0.043397559	0.163306252	0.766029358
SMAD7	SMAD7	0.082419991	0.386966056	0.921875
STAT2	STAT2	0.118699561	0.305693619	0.86504364
SOX8	SOX8	0.140480135	0.252478558	0.322265625
Pik3ca	Pik3ca	0.148040175	0.429949717	0.625
SOCS4	SOCS4	0.182680673	0.073114042	0.125
TLR-7	TLR-7	0.230531217	0.301068758	0.76953125
SOX14	SOX14	0.243463776	0.767162296	0.10546875
GFP	GFP	0.274589486	0.245691589	0.064453125
IFI16 (ifi204)	IFI16 (ifi204)	0.287719048	0.426333628	0.083984375
NFATp(NFATc2)	NFATp(NFATc2)	0.306958124	0.426753592	0.193359375
TICAM2 (TRAM)	TICAM2 (TRAM)	0.317433812	0.427577136	0.4921875
Ets-1	Ets-1	0.344068292	0.200013942	0.275390625
TLR-8	TLR-8	0.375634398	0.549707386	0.064453125
RIG-I (DDX58)	RIG-I (DDX58)	0.454210328	0.82980994	0.375
TAK-1 (Map3k7)	TAK-1 (Map3k7) *	0.469875871	0.322917234	0.037109375
SREBf1	SREBf1	0.531164413	1.431990866	0.84375
IRF2	IRF2 *	0.584258857	0.283681704	0.00025177
MyD88	MyD88	0.597376684	0.3836066	0.064453125
EGR-3	EGR-3 *	0.664121689	0.528425984	0.001953125
SOX1	SOX1 *	0.668740738	0.684472969	0.013671875
Ets-2	Ets-2 *	0.722953557	0.315290939	0.009765625
NFATc(NFATc1)	NFATc(NFATc1) *	0.8308325	0.534829474	0.001953125
SOX3	SOX3	0.899843414	0.615137904	0.064453125
Insig2	Insig2	1.086390315	1.519116198	1
IRF9	IRF9	1.170316618	0.429223994	0.083984375
IRF3	IRF3	1.316896666	0.983282679	0.522613525
SOX9	SOX9 *	1.576562264	0.910664942	0.009765625
TLR-4	TLR-4 *	1.850553929	0.688654484	0.001953125
TBK-1	TBK-1 *	1.874451907	0.324103047	0.001953125

gLuc-assay	siglabels *=significant	sirna.median	sirna.sem	p-value (wilcoxon signed rank test)
TLR-7	TLR-7	-0.978465183	0.69352088	0.5
SOCS3	SOCS3	-0.941866944	0.112076869	0.25
CIP21	CIP21	-0.932208204	0.340927028	0.25
SOCS7	SOCS7	-0.894035315	0.155004946	0.25
gaussia Luciferase	gaussia Luciferase *	-0.833721335	0.285785992	0.03125
YY1	YY1 *	-0.790896554	0.305111369	0.015625
IRAK-4	IRAK-4	-0.754959405	0.27886255	0.25
YAF2	YAF2 *	-0.731020441	0.213574121	0.015625
SOX13	SOX13	-0.711654832	0.535954178	0.5
IRAK-1	IRAK-1	-0.709916116	0.51747075	0.5
TIRAP (Mal)	TIRAP (Mal)	-0.692845583	0.356165577	0.5
ELK1	ELK1 *	-0.687040717	0.442162817	0.046875
AIM2	AIM2	-0.673253325	0.287680282	0.25
Sp1	Sp1 *	-0.673173193	0.338702912	0.03125
SRF	SRF *	-0.660627787	0.210001886	0.015625
IRF6	IRF6 *	-0.65097742	0.311843119	0.03125
MyD88	MyD88	-0.640581385	0.286551984	0.25
RIG-I (DDX58)	RIG-I (DDX58)	-0.640251484	0.427288292	0.5
TLR-9	TLR-9	-0.611237469	0.423337239	0.5
SMAD7	SMAD7	-0.602665872	0.283859496	0.5
TBP	TBP *	-0.60130929	0.314285793	0.03125
RXRA	RXRA *	-0.59652058	0.278021434	0.046875
SP7	SP7 *	-0.587918075	0.314823551	0.015625
non Targenting pool#1	non Targenting pool#1 *	-0.575676158	0.177535758	0.046875
SOCS5	SOCS5	-0.569122643	0.21257399	0.25
JUND1	JUND1	-0.557874421	0.385147142	0.21875
FOS	FOS	-0.556655663	0.575418967	0.078125
MKP-1 (DUSP1)	MKP-1 (DUSP1)	-0.552329501	0.803201993	0.25
IRAK-2	IRAK-2	-0.543476099	0.30482386	0.5
SOX14	SOX14	-0.534703695	0.366176423	0.5
NFKBIB	NFKBIB	-0.532634884	0.211443752	0.125
SOX1	SOX1	-0.5176835	0.255385742	0.25

RELA	RELA *	-0.515254237	0.226307801	0.046875
TFDp1/Dp1/DrTF1	TFDp1/Dp1/DrTF1	-0.480684493	0.1549676	0.25
RNF139	RNF139	-0.464552682	0.40298116	0.25
ELK4	ELK4 *	-0.453794434	0.16915747	0.015625
IRF3	IRF3 *	-0.435211636	0.129459165	0.015625
GSK3b	GSK3b	-0.433524789	0.23930281	0.25
TICAM2 (TRAM)	TICAM2 (TRAM)	-0.432499588	0.334995168	0.5
TLR-2	TLR-2	-0.42757145	0.238606788	0.5
Ets-1	Ets-1	-0.421660235	0.497432897	0.75
SOCS4	SOCS4	-0.412507475	0.486305731	0.5
Stat4	Stat4	-0.412350098	0.280458685	0.15625
SARM1	SARM1	-0.404248641	0.141053513	0.25
CEBPb	CEBPb	-0.39589374	0.626428294	0.75
SP6	SP6 *	-0.384659813	0.221300986	0.046875
E2F1	E2F1	-0.381524913	0.250486846	0.15625
PTGS2 (Cox2)	PTGS2 (Cox2)	-0.379754082	0.193936202	0.5
Nfy-beta	Nfy-beta	-0.374473401	0.51144	0.75
SOX6	SOX6	-0.368820827	0.385458781	0.5
CdK2	CdK2	-0.367075697	0.182191504	0.25
IRF7	IRF7	-0.361807006	0.236396858	0.078125
SOX7	SOX7	-0.360748396	0.355121704	0.75
TLR-8	TLR-8	-0.359414922	0.356843491	0.5
MEKK1	MEKK1	-0.35285555	0.859526627	0.25
IRF8	IRF8	-0.347491563	0.123282462	0.25
Stat6	Stat6	-0.345474451	0.416129656	0.109375
TICAM1 (TRIF)	TICAM1 (TRIF)	-0.344535199	0.107875708	0.25
IRF5	IRF5 *	-0.333845169	0.227963585	0.015625
SMAD3	SMAD3	-0.331523345	0.476998887	0.75
SOX12	SOX12	-0.320785477	0.302279571	0.5
SPI-B	SPI-B	-0.319275102	0.569499706	1
TLR-4	TLR-4	-0.314242152	0.115454341	0.25
FOSB	FOSB *	-0.312963155	0.370566328	0.03125
SP4	SP4	-0.301876633	0.319412951	0.078125
TRAF6	TRAF6	-0.30122096	0.31130205	0.5
IRF4	IRF4 *	-0.30065764	0.164286036	0.015625
SOX8	SOX8	-0.298718693	0.281530392	0.5
SOX15	SOX15	-0.294360421	0.677134915	1
NFKB1	NFKB1	-0.283538912	0.161079024	0.21875
STAT5A	STAT5A	-0.283167449	0.175765955	0.15625
Nfy-gamma	Nfy-gamma	-0.282794682	0.447354487	1
RELB	RELB *	-0.282233867	0.13316894	0.03125
NFAT4(NFATc3)	NFAT4(NFATc3)	-0.278291069	0.278131544	0.25
CITED2	CITED2	-0.275008868	0.309376522	0.21875
RARB	RARB	-0.274958025	0.159724746	0.15625
SOX11	SOX11	-0.274055162	0.166028397	0.5
STAT3	STAT3	-0.272332806	0.148320325	0.109375
CITED1	CITED1	-0.271442283	0.41497435	0.296875
EGR-3	EGR-3	-0.264499055	0.385422124	0.75
IE3	IE3	-0.264297505	0.235671437	0.46875
SOCS1	SOCS1	-0.263711417	0.084713757	0.25
IRF9	IRF9	-0.262653899	0.112292517	0.25
RXRG	RXRG	-0.255080713	0.338384764	0.578125
M54	M54	-0.250796532	0.349641969	0.21875
STAT5B	STAT5B	-0.2495636	0.325927402	0.46875
STAT1	STAT1 *	-0.24926218	0.170339097	0.046875
NFAT3(NFATc4)	NFAT3(NFATc4)	-0.247846945	0.298222031	0.75
SOX18	SOX18	-0.242718266	0.480456552	1
TBK-1	TBK-1	-0.242456913	0.205284211	0.5
IRF1	IRF1	-0.233440406	0.110946241	0.21875
SOX2	SOX2	-0.228176065	0.025641479	0.25
IRAK-3	IRAK-3	-0.227846128	0.41596753	0.5
RARA	RARA	-0.227390012	0.157915115	0.15625
SOX5	SOX5	-0.223742948	0.254200487	0.5
TAK-1 (Map3k7)	TAK-1 (Map3k7)	-0.22213567	0.148253476	0.25
FOSL2	FOSL2	-0.217183231	0.282423373	0.078125
TLR-3	TLR-3	-0.215117932	0.337702118	0.75
STAT2	STAT2	-0.207975319	0.168085187	0.15625

IRF2	IRF2	-0.204989655	0.256757108	0.109375
NFKB2	NFKB2	-0.20428264	0.230500584	0.46875
ELK3	ELK3	-0.20368829	0.251308027	0.078125
RISC	RISC	-0.203109897	0.22708157	0.322265625
Fosl1	Fosl1	-0.194385619	0.19017396	0.15625
Ets-2	Ets-2	-0.192237956	0.574017363	1
SOX4	SOX4	-0.189957513	0.098250121	0.25
SOCS6	SOCS6	-0.181967473	0.38316961	0.5
CITED4	CITED4	-0.174477162	0.244389053	0.46875
EGR-1	EGR-1	-0.173332491	0.555625294	1
LITAF(PIG7)	LITAF(PIG7)	-0.164341802	0.240577289	0.75
ATF4	ATF4	-0.154066042	0.42823031	0.109375
CEBPg	CEBPg	-0.146537337	0.552461265	1
NFATc(NFATc1)	NFATc(NFATc1)	-0.142797595	0.286086691	1
NFkBIB	NFkBIB	-0.133100862	0.226564843	1
RXRB	RXRB	-0.130301906	0.320363472	0.578125
SP2	SP2	-0.122641581	0.217751917	0.375
RARG	RARG	-0.11631141	0.202713453	0.109375
Pu.1 (SPI-1)	Pu.1 (SPI-1)	-0.101600914	0.373153099	1
CREBL1	CREBL1	-0.093402091	0.240032901	0.578125
JUNB	JUNB	-0.089463196	0.361731865	0.9375
CEBPz	CEBPz	-0.087158362	0.60535919	1
ZBP-1	ZBP-1	-0.077342998	0.287328002	1
CREB3L1	CREB3L1	-0.073053513	0.314019004	0.375
SOX30	SOX30	-0.061620928	0.41594968	1
IFI16 (ifi204)	IFI16 (ifi204)	-0.05923738	0.189284623	0.75
Daxx	Daxx	-0.055727314	0.361284047	1
ATF5	ATF5	-0.052473575	0.233736762	0.296875
Pik3ca	Pik3ca	-0.048922025	0.383804725	1
SP3	SP3	-0.041012907	0.354119525	0.578125
CdK1	CdK1	-0.026910592	0.289349418	0.75
Nfy-alpha	Nfy-alpha	-0.012763799	0.173799243	1
GFP	GFP	-0.009772756	0.511767188	1
SOX10	SOX10	-0.007227824	0.146543032	0.75
ATF6	ATF6	-0.001920111	0.239368158	0.8125
SOX21	SOX21	-0.00146614	0.211689255	1
ATF3	ATF3	0.011734715	0.152459745	0.8125
SOX3	SOX3	0.016377854	0.225366689	0.75
CREB3	CREB3	0.017332582	0.1244406	0.9375
EP300	EP300	0.027007223	0.245905263	0.8125
SOX9	SOX9	0.05295582	0.221718615	0.75
CEBPa	CEBPa	0.054209427	0.853756641	0.75
SOX17	SOX17	0.0705396	0.413009314	0.5
MDA-5 (IFih1)	MDA-5 (IFih1)	0.073570228	0.134574269	1
CREB1	CREB1	0.081669281	0.179392032	1
CREB3L4	CREB3L4	0.089673386	0.255974598	0.9375
JUNDM2	JUNDM2	0.092958131	0.391089195	0.578125
NFAT5(TonEBP)	NFAT5(TonEBP)	0.134014523	0.071121567	0.5
NFATp(NFATc2)	NFATp(NFATc2)	0.141930249	0.096618767	0.5
TP53	TP53	0.146239539	0.229290399	0.75
ATF1	ATF1	0.156301592	0.169147438	0.296875
EGR-2	EGR-2	0.168811538	0.531722406	0.25
SOCS2	SOCS2	0.185600505	0.395341463	1
CREBBP	CREBBP	0.242543804	0.185756605	0.375
JUN	JUN	0.247349146	0.222511066	0.15625
ATF2	ATF2	0.290609858	0.180450101	0.296875
REL	REL	0.310770186	0.235044943	0.46875
ATF7	ATF7	0.32518958	0.269605222	0.375
