# Supplementary material for: A Temporal Gate for Viral Enhancers to Co-opt Toll-Like-Receptor Transcriptional Activation Pathways upon Acute Infection
Source: PLoS Pathog. 2015 Apr 9;11(4):e1004737. doi: 10.1371/journal.ppat.1004737 (PMC4391941; doi:10.1371/journal.ppat.1004737)
Supplement: S4 Table — List of replicates distribution per siRNA screen used for statistical Meta-analysis. (PDF) [file ppat.1004737.s014.pdf]

GFP screen	S1-8	S9-20	S21-24
siRNA knock-down replicates (per siRNA)	4	2	2
infected controls	32	16	32

gLuc screen	S1	S2-4	S5	S6	S7	S8	S9	S10
siRNA knock-down replicates (per siRNA)	3	3	1	2	2	1	2	2
infected controls	21	24	8	16	32	8	16	32
