# Supplementary material for: A Temporal Gate for Viral Enhancers to Co-opt Toll-Like-Receptor Transcriptional Activation Pathways upon Acute Infection
Source: PLoS Pathog. 2015 Apr 9;11(4):e1004737. doi: 10.1371/journal.ppat.1004737 (PMC4391941; doi:10.1371/journal.ppat.1004737)
Supplement: S5 Table — Positions of binding motifs in the human CMV enhancer are given relative to transcription start site of Ie1. Point mutations are shown and where applicable introduced endonuclease restriction sites are indicated by name of the enzyme. (PDF) [file ppat.1004737.s015.pdf]

NFkB mutations: REF [50]

mutation NFkB 1:	wt	5’$_{-422}$G**G**GA**CT**T**TC**C$_{-413}$ 3’	
	mut	G**A**GA**TC**T**GG**C	*Bgl* II
mutation NFkB 2:	wt	5’$_{-271}$G**G**GA**CT**T**TC**C$_{-262}$ 3’	
	mut	GAGATCTGGC	*Bgl* II
mutation NFkB 3:	wt	5’$_{-165}$GGGATTTCC$_{-157}$ 3’	
	mut	G**TACC**TT**G**C	*Kpn* I
mutation NFkB 4:	wt	5’$_{-103}$**G**GG**A**CTTT**CC**$_{-94}$ 3’	
	mut	**A**GG**C**CTTT**GG**	*Stu* I

ATF mutations

mutation ATF 1:	wt	5’$_{-464}$T**T**GA**CG**TC$_{-457}$	
	mut	T**A**GA**TC**TC	*Bgl* II
mutation ATF 2:	wt	5’$_{-410}$TG**ACGT**CA$_{-403}$	
	mut	TG**GTAC**CA	*Kpn* I
mutation ATF 3:	wt	5’$_{-328}$T**T**G**A**C**G**TC$_{-321}$	
	mut	T**A**G**G**C**C**TC	*Stu* I
mutation ATF 4:	wt	5’$_{-142}$T**TG**A**CGT**C$_{-135}$	
	mut	T**GA**A**TTC**C	*Eco* RI
mutation ATF 5:	wt	5’$_{-66}$GA**CGCA**AA$_{-59}$	
	mut	GA**ATTC**AA	*Eco* RI

AP1 mutations: REF [69]

mutation Ap1 1:	wt	5’$_{-239}$TTA**GTCATC**$_{-231}$ 3’	
	mut	TTA**AGATCT**	*Bgl* I
mutation Ap1 2:	wt	5’$_{-174}$**T**G**A**C**T**CA$_{-168}$ 3’	
	mut	**G**G**G**C**C**CA	*Apa* I

RARE mutations: REF [76]

mutation RARE A:	wt	5’$_{-557}$GGG**TC**ATTAG**T**TC**A**$_{-544}$ 3’	
	mut	GGG**AA**ATTAG**A**TC**T**	*Bgl* II
mutation RARE B:	wt	5’$_{-488}$T**GA**CCGCCCAA**C**G**A**CCC$_{-472}$ 3’	
	mut	T**CT**CCGCCCAA**G**G**G**CCC	*Apa* I
mutation RARE C:	wt	5’$_{-291}$TG**CC**CAGTACATG**AC**CT$_{-275}$ 3’	
	mut	TG**TT**CAGTACATG**TT**CT	
mutation RARE D:	wt	5’$_{+34}$T**GA**CCTCCATAGAAGACAC$_{+52}$ 3’	
	mut	T**CT**CCTCCATAGAAGACAC	
